# Microbial Conjugation Studies of Licochalcones and Xanthohumol

**DOI:** 10.3390/ijms22136893

**Published:** 2021-06-26

**Authors:** Fubo Han, Yina Xiao, Ik-Soo Lee

**Affiliations:** College of Pharmacy, Chonnam National University, Gwangju 61186, Korea; hanfubo0306@gmail.com (F.H.); yogurtxiao@163.com (Y.X.)

**Keywords:** licochalcones, xanthohumol, sulfation, microbial conjugation

## Abstract

Microbial conjugation studies of licochalcones (**1**–**4**) and xanthohumol (**5**) were performed by using the fungi *Mucor hiemalis* and *Absidia coerulea*. As a result, one new glucosylated metabolite was produced by *M. hiemalis* whereas four new and three known sulfated metabolites were obtained by transformation with *A. coerulea*. Chemical structures of all the metabolites were elucidated on the basis of 1D-, 2D-NMR and mass spectroscopic data analyses. These results could contribute to a better understanding of the metabolic fates of licochalcones and xanthohumol in mammalian systems. Although licochalcone A 4′-sulfate (**7**) showed less cytotoxic activity against human cancer cell lines compared to its substrate licochalcone A, its activity was fairly retained with the IC_50_ values in the range of 27.35–43.07 μM.

## 1. Introduction

The licorice root, known as “*Radix Glycyrrhizae*”, has been used in traditional Chinese medicines for centuries to treat respiratory infections, gastritis, tremors, and peptic ulcers [1,2]. At present, there are hundreds of compounds isolated from licorice, including flavonoids, triterpene saponins, and alkaloids which are responsible for antidiabetic, neuroprotective, anti-inflammatory, and other bioactive effects [2]. Licochalcones, the significant components of licorice flavonoids, including licochalcone A (LCA, **1**), licochalcone B (LCB, **2**), licochalcone C (LCC, **3**), and so on, were reported to exhibit a variety of bioactivities, and can be used in food and cosmetic industries [2]. In recent years, licochalcones have attracted more attention from research communities due to their anticancer potential against different kinds of cancers, such as breast, lung, and gastric cancers [2,3]. Although the pharmacological activities of licochalcones have been extensively investigated, the metabolic pathway of licochalcones in mammals remains largely unknown. It has been reported that LCA can undergo both phase I and phase II metabolic processes in vitro, with the formation of oxygenated and glucuronidated metabolites [4,5], and the in vivo metabolism studies of LCA led to the determination of glucuronidated, *N*-acetyl-l-cysteine conjugated, and sulfated metabolites [5,6].

**Figure 1:**
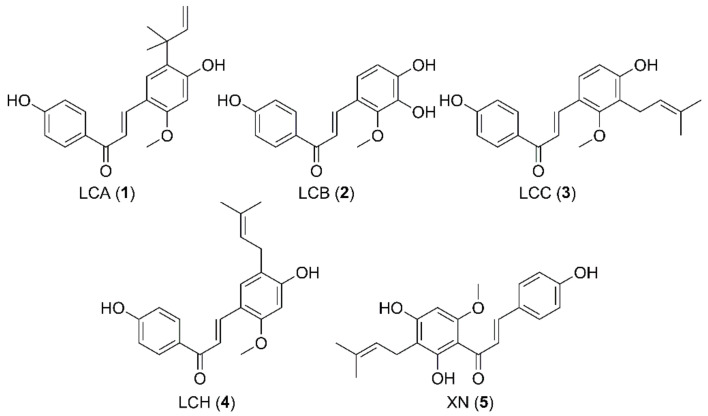
Chemical structures of licochalcones A–C (**1**–**3**), H (**4**) and xanthohumol (**5**).

In comparison to licochalcones, metabolism of xanthohumol (XN, **5**) (Figure 1), a prenylated chalcone from hops, was quite extensively examined and both in vitro and in vivo studies have been conducted. The studies using human and rat liver microsomes resulted in the identification of hydroxylated, cyclized, and glucuronidated metabolites [7,8,9]. In addition, sulfate conjugated metabolites of XN were observed after using sulfotransferases and detected in rats after administration of hop extract [10,11]. However, it seems impossible to identify the conjugation position of the sulfate group by using the LC/MS analysis method without a respective reference compound. It was reported that the phase II enzymes UDP-glucuronosyltransferases and sulfotransferases were not only found in liver but also in other tissues [12], suggesting that LCA and XN could be also metabolized by other organs and tissues.

LCA and XN undergo complex biotransformation processes in mammalian systems and they are mainly metabolized by phase II enzymes, although their interactions with cytochrome P450 enzymes cannot be excluded. Polyphenolic substances are easy targets for conjugation reactions and these are indeed the major biotransformation pathways of licochalcones [13]. Therefore, it is more relevant to investigate the effects of metabolically conjugated derivatives on human health rather than their parent compounds [14]. Generally, sulfate conjugation is considered as a detoxification pathway for endogenous and exogenous phenolic compounds, as the conjugated derivatives are polar and water-soluble, which facilitates their elimination from the body [15,16]. Nevertheless, it is clear today that detoxification is not the only function of sulfation, and the biological activities of compounds after sulfation can be retained, lowered, abolished, or even increased [14,17,18]. For instance, mangostin 3-sulfate exhibited stronger anti-mycobacterial activity against *Mycobacterium tuberculosis* than α-mangostin [19]. However, compared to the glucuronidated and methylated conjugates, the biological properties and cellular activities of sulfated conjugates were least studied [14]. It is important to expand the structural diversity of sulfated derivatives via chemical or biological methods for biological studies.

Microbial transformation studies are regarded as one of the useful in vitro models to mimic and predict the mammalian metabolism process of xenobiotic compounds [20]. In addition, it allows the selective conversion of compounds into derivatives, which are difficult to obtain in chemical synthesis under mild conditions [21]. Such processes can provide sufficient amounts of metabolites for structure elucidation and pharmacological activity evaluation [21]. Khan and colleagues have reported that *Absidia glauca* [22], *Aspergillus alliaceus* [23], *Beauveria bassiana* [24], *Chaetomium cochlioides*, *Cunninghamella blakesleeana* [23], *Cunninghamella echinulata* [25], *Mortierella zonata* [23], and *Mucor ramannianus* [26] could transform the flavones and flavanones to their corresponding sulfated metabolites. Thus, biotransformation conducted by microorganisms could be used as a simple and efficient method for obtaining not only phase I but also phase II metabolites of chalcones [20].

To the best of our knowledge, no metabolism studies of licochalcones B–D and H were performed so far, except the study previously carried out in this lab [27]. Thus, in order to identify the conjugated metabolites of licochalcones, further studies need to be performed. In the present study, microbial transformation of licochalcones A–C, H and xanthohumol (**1**–**5**) was carried out with *Mucor hiemalis* and *Absidia coerulea*, affording one new glucosylated and four new sulfated metabolites, respectively. The conjugated metabolites could be used as authentic reference standards in metabolic studies of licochalcones and xanthohumol.

## 2. Results and Discussion

### 2.1. Microbial Transformation of Licochalcone A and Xanthohumol by M. hiemalis

During our previous study, it was observed that licochalcones B, C, H could be *O*-glucosylated by using the fungus *M. hiemalis* [27]. Here, the same process was performed with LCA and XN, which led to the isolation of a new compound licochalcone A 4-*O*-β-d-glucopyranoside (**6**) together with a known compound xanthohumol 4′-*O*-β-d-glucopyranoside [28]. Yin and colleagues discovered a phenolic glycosyltransferase MhGT1 from *M. hiemalis* and applied the enzymatic approach to obtain a series of glycosylated phenolic compounds [29]. Likewise, the *O*-glucosylation process observed here was speculated to be catalyzed by the enzyme glycosyltransferase formed by *M. hiemalis*.

**Figure 2:**
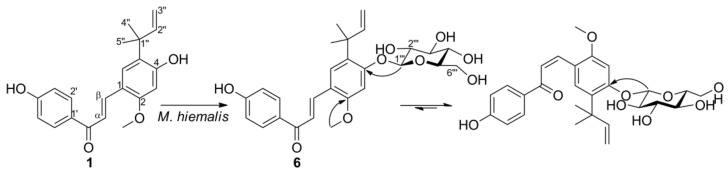
Microbial transformation of **1** by *M. hiemalis*. Selected HMBC correlations (^1^H→^13^C) of metabolite **6** are indicated by arrows.

Compound **6** was isolated as an orange amorphous powder. HRESIMS of **6** exhibited a quasi-molecular ion at *m/z* 523.1943 [M+Na]^+^ (calcd. for C_27_H_3__2_O_9_Na, 523.1944), suggesting the molecular formula of **6** as C_27_H_32_O_9_, which was one glucose unit higher than that of **1**. The presence of sugar moiety was further confirmed by comparison of its NMR spectra with those of **1**, which showed an anomeric proton signal at δ_H_ 5.01 (1H, d, *J* = 7.0 Hz) in ^1^H-NMR spectrum and a hexose carbon signals at δ_C_ (100.5, 73.8, 77.8, 70.7, 78, and 61.4) in ^13^C-NMR. The significant downfield-shifted proton signal of H-3 in ring B indicated that the glucose moiety was attached to 4-OH. HMBC correlation from the anomeric proton signal at δ_H_ 5.01 (H-1′′′) to the carbon signal at δ_C_ 159.4 (C-4) confirmed the assignment of glycosylation at C-4 (Figure 2). The resonances of the glucose moiety were assigned by HSQC and HMBC experiments (See Supplementary Materials). TLC comparison with the authentic sample after acid hydrolysis of **6** led to the elucidation of the glucose moiety to be d-form [28,30]. The aglycone part of the ^1^H- and ^13^C-NMR spectra of **6** revealed two sets of signals in the integral ratio of around 1:1.3. Based on the two pairs of H-α and H-β proton signals at δ_H_ 7.67 (1H, d, *J* = 15.7 Hz) and 7.92 (1H, d, *J* = 15.7 Hz), together with δ_H_ 6.44 (1H, d, *J* = 12.8 Hz) and 7.03 (1H, d, *J* = 12.8 Hz), **6** was clearly assigned as a mixture of *trans-* and *cis*-isomers. Based on the above data and extensive 2D NMR experiments, structure of the compound **6** was assigned as licochalcone A 4-*O*-β-d-glucopyranoside.

### 2.2. Microbial Transformation of Licochalcones A, B, C, H, and Xanthohumol by A. coerulea

Screening of licochalcones and XN (**1**–**5**) with several microorganisms showed that only *A. coerulea* was able to produce the highly polar metabolites, more polar than the metabolites transformed by *M. hiemalis*. During the screening procedures, it was also observed that by using more sulfate-containing media, the yield of sulfate-conjugated metabolites could be increased. Thus, instead of using the original incubation medium suggested by KCTC, the scale-up fermentation process was performed by using a modified Czapek Dox media (dextrose 10 g/L, sodium nitrate 2 g/L, dipotassium phosphate 1 g/L, magnesium sulfate 0.5 g/L, potassium chloride 0.5 g/L, ferrous sulfate 0.02 g/L). Isolation of the metabolites from large-scale fermentations was processed successfully by using EtOAc as extraction solvent.

**Figure 3:**
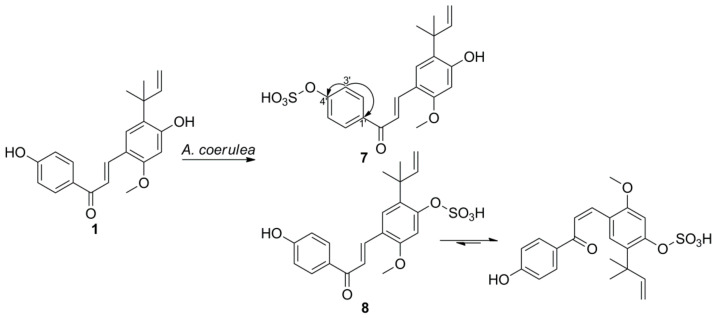
Microbial transformation of **1** by *A.* *coerulea*. Selected HMBC correlations (^1^H→^13^C) of metabolite **7** are indicated by arrows.

Metabolite **7** was isolated as a yellow amorphous powder. The UV spectrum showed maximum absorption bands at 266 and 387 nm, similar to those of substrate **1**. The HRESIMS spectra of **7** displayed a molecular ion peak at *m/z* 417.1024 [M-H]^−^ and a fragment ion peak at *m/z* 337.1449 [M-H-80]^−^, suggesting the presence of a sulfate group. In addition, the isotopic mass peak at *m/z* 419.1087 [M-H+2]^−^ indicated the presence of a sulfur atom in the compound. The IR spectrum of **7** showed the presence of two strong bands corresponding to the S=O (1251 cm^−1^) and C-O-S (1050 cm^−^^1^), supporting the presence of a sulfate group [31]. The presence of the sulfate group was further supported by the formation of white precipitate after treating the aqueous layer of acid-hydrolyzed **7** with BaCl_2_ [31]. Comparison of the ^1^H- and ^13^C-NMR spectroscopic data of **7** with **1** revealed the resonance signals being almost identical in both compounds, except for the downfield shift of H-3′/5′, C-1′, C-3′/5′ (0.44, 4.0, and 4.7 ppm, respectively) and the upfield shift of C-4′ (4.4 ppm), implying that the sulfate moiety was conjugated with 4′-OH. These shifts were consistent with naringenin 4′-sulfate, a transformed product of naringenin, because the carbon directly attached to the sulfate ester and the carbons in the *meta*-position are upfield-shifted, and the protons and carbons in *ortho-* and *para*-positions to the sulfate group are downfield-shifted [18,32]. The sulfate position at C-4′ was further confirmed by HMBC correlations of H-3′/5′ with C-1′ and C-4′ (Figure 3). Therefore, the structure of **7** was identified as licochalcone A 4′-sulfate.

Metabolite **8** was obtained as a mixture with **7**. In the ^1^H-NMR spectrum of **8**, the significant downfield-shifted signal at δ_H_ 7.51 (H-3) was observed, indicating that the sulfate group was attached to 4-OH. Two sets of proton resonance signals were observed for **8**, which is similar to the glucosylated metabolite **6**. Thus, the structure of **8** was identified as *trans-* and *cis-*isomers of licochalcone A 4-sulfate.

**Figure 4:**
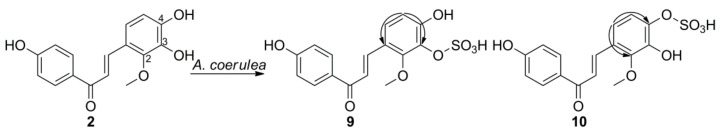
Microbial transformation of **2** by *A.* *coerulea*. Selected HMBC correlations (^1^H→^13^C) of metabolites **9** and **10** are indicated by arrows.

Metabolite **9** was obtained as a yellow solid. The HRESIMS spectra of **9** exhibited a molecular ion peak at *m/z* 365.0336 [M-H]^−^ and a fragment ion peak at *m/z* 285.0767 [M-H-80]^−^, suggesting the presence of a sulfate group. The ^1^H-NMR data of **9** were similar to **2**, except for the H-6 signal, which was downfield-shifted by 0.33 ppm. Comparison of ^13^C-NMR data of **9** and substrate **2** showed that the C-3 signal was upfield-shifted by 5.7 ppm, and the signals of C-2, C-4, and C-6 were downfield-shifted by 4.1, 3.2, and 5.0 ppm, respectively [33]. All of these suggested that the sulfate group was attached to 3-OH, and this was further confirmed by the HMBC correlations between H-5 and C-1/3 (Figure 4). Thus, the structure of **9** was identified as licochalcone B 3-sulfate.

Metabolite **10** was obtained as a mixture with **9**. In the ^1^H-NMR spectrum of **10**, the significant downfield-shifted signal at δ_H_ 7.22 (H-5) was observed, indicating that the sulfate group was attached to 4-OH. This was supported by the deshielding of C-3 and C-5 carbons, which are *ortho* to the sulfate site, compared to those of substrate **2** in the ^13^C NMR of **10** by 3.0 and 5.2 ppm, respectively. Furthermore, the *para* carbon at C-1 in **10** was deshielded by 4.0 ppm, while the *ipso* carbon at C-4 position was shielded by 7.7 ppm [33]. Based on the above analysis, the structure of **10** was identified as licochalcone B 4-sulfate. The resonances of **10** were completely assigned by HSQC and HMBC spectra (See Supplementary Materials).

**Figure 5:**
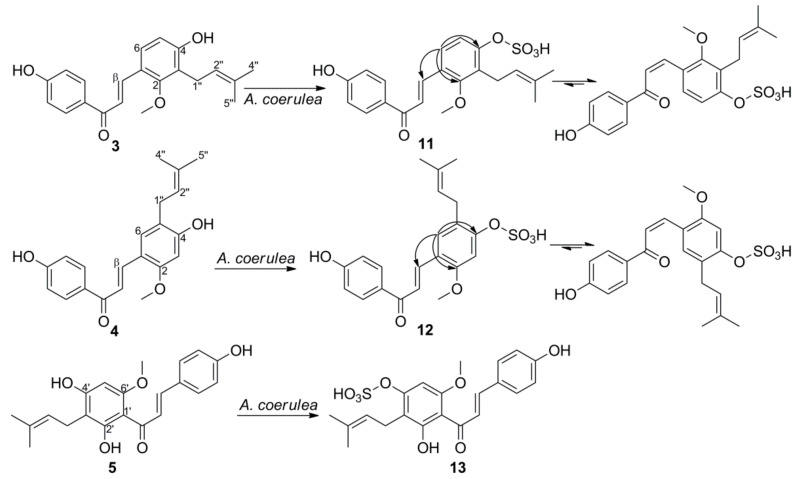
Microbial transformation of **3**–**5** by *A.* *coerulea*. Selected HMBC correlations (^1^H→^13^C) of metabolites **11** and **12** are indicated by arrows.

Metabolite **11** was obtained as a yellow solid. The HRESIMS spectra of **11** exhibited a molecular ion peak at *m/z* 417.1010 [M-H]^−^ and a fragment ion peak at *m/z* 337.1438 [M-H-80]^−^, suggesting the presence of a sulfate group. Two sets of signals in the integral ratio of around 1:1.1 were observed in the ^1^H- and ^13^C-NMR spectra of **11**. It was assigned as a mixture of *trans-* and *cis*-isomers according to the two pairs of H-α and H-β proton signals at δ_H_ 7.73 (1H, d, *J* = 15.9 Hz) and 8.01 (1H, d, *J* = 15.9 Hz), together with δ_H_ 6.65 (1H, d, *J* = 12.9 Hz) and 7.10 (1H, d, *J* = 12.9 Hz). Comparison of the ^1^H-NMR spectrum of LCC sulfate **11** with the parent compound **3** clearly showed a strong difference in the chemical shift for H-5 in ring B, whereas the chemical shifts of protons in ring A were only slightly changed, suggesting that the sulfate moiety was attached to 4-OH. This was supported by the significant upfield-shifted signal of C-4 and downfield-shifted signals of C-1, C-3, and C-5 in the ^13^C-NMR spectra of **11** compared with those of **3** [33]. The HMBC correlations between H-6 and C-4, C-2 further confirmed the attachment at C4-OH (Figure 5). Thus, the structure of **11** was identified as licochalcone C 4-sulfate.

Metabolite **12** was obtained as a yellow solid. The HRESIMS spectra of **12** exhibited a molecular ion peak at *m/z* 417.0974 [M-H]^−^ and a fragment ion peak at *m/z* 96.9881, suggesting the presence of a sulfate group. Two sets of signals in the integral ratio of around 1:1.2 were observed in the ^1^H- and ^13^C-NMR spectra of **12**. It was assigned as a mixture of *trans*- and *cis*-isomers, according to the two pairs of H-α and H-β proton signals at δ_H_ 7.68 (1H, d, *J* = 15.8 Hz) and 7.98 (1H, d, *J* = 15.8 Hz), together with δ_H_ 6.43 (1H, d, *J* = 12.8 Hz) and 7.12 (1H, d, *J* = 12.8 Hz). Comparison of the ^1^H-NMR spectrum of LCH sulfate **12** with the parent compound **4** clearly showed a strong difference in the chemical shift for H-3 in ring B, whereas the chemical shifts of protons in ring A were only slightly changed, suggesting that the sulfate moiety was attached to 4-OH. This was supported by the significant upfield-shifted signal of C-4 and downfield-shifted signals of C-1, C-3, and C-5 in the ^13^C-NMR spectra of **12** compared with those of **4** [34]. Thus, the structure of **12** was identified as licochalcone H 4-sulfate. The resonances of **12** were completely assigned by HSQC and HMBC spectra (See Supplementary Materials).

Metabolite **13** was isolated as a yellow amorphous powder. The HRESIMS spectra of **13** displayed a molecular ion peak at *m/z* 433.1092 [M-H]^−^ and a fragment ion peak at *m/z* 353.1355 [M-H-80]^−^, suggesting the presence of a sulfate group. Comparison of the ^1^H- and ^13^C-NMR spectroscopic data of **13** with **5** indicated almost identical resonances for both compounds, except for the downfield-shift of H-5′, C-1′, C-3′, C-5′ (0.88, 3.2, 6.0, and 4.7 ppm, respectively) and the upfield-shift of C-4′ (5.8 ppm), implying that the sulfate moiety was conjugated with 4′-OH [35]. Therefore, the structure of **13** was identified as xanthohumol 4′-sulfate.

**Figure 6:**
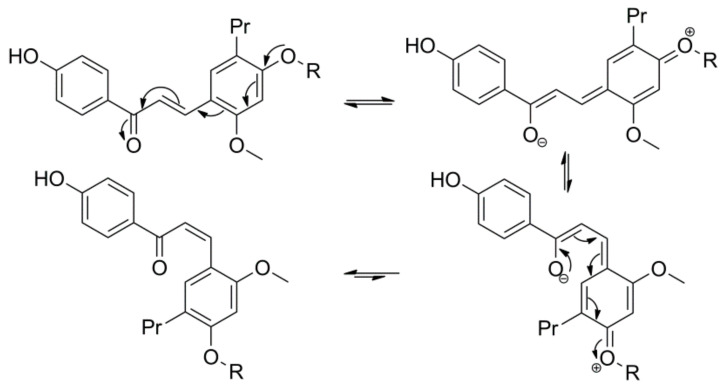
The proposed mechanism of *trans*-*cis* isomerization.

It has been reported that chalcones can undergo *trans* to *cis* transformation in solution when light exposure is available. The presence of a free hydroxyl group at the C-4 position of chalcones inhibits photoisomerization between the *trans-* and *cis-*isomers, as the hydroxyl group at C-4 position enables keto-enol tautomerization and free rotation around the α,β-bond, which allows the rapid conversion of *cis* chalcones to the stable *trans*-isomers [4]. The electron-donating group at C-4 position is expected to weaken the α,β-double bond of chalcones through electron-delocalization effects (Figure 6), thus the electron-withdrawing effects carried out by the glucose and sulfate moieties of **6**, **8**, **11,** and **12** may quench the electron-donating capacity of the *O*-atom at C-4 [36]. As a result, glucosylation and sulfation of C4-OH raise the barrier and make the *cis*-*trans* chalcone isomerization a slow kinetically distinct process [36]. As demonstrated with the isolated metabolites **6**, **11**, and **12**, the ratio of *cis-* to *trans-*isomers was found to be >1 in the NMR solvent, suggesting that the *cis* isomer produced here is structurally stable under the observed conditions [37]. Such observations are quite remarkable in this case as it is commonly known that *trans* chalcones are more stable than their *cis*-isomers.

The conjugation reactions are widely known as major metabolic pathways for the detoxification of xenobiotic compounds [15]. The conjugated derivatives are more polar and water-soluble and therefore more easily excreted from the body through urine or feces [14,38]. However, an increasing amount of evidence suggests that detoxification is not the only role of sulfation in the organisms. Sulfation might change the biological activities of numerous compounds, and some of the sulfated compounds work therapeutically against cancer, diabetes, and various metabolic diseases [18,39]. A demonstration of parallelism between mammalian and microbial systems, conjugation reaction is of considerable importance for the drug metabolism investigations. However, sulfation in microbial systems is rare and reports on this topic are limited. So far, in addition to the list of microorganisms reported by Khan [22,23,24,25,26], several fungi including *Absidia coerulea* [40], *Colletotrichum gloeosporioides* [19], *Cunninghamella elegans* [32], *Gliocladium deliquescens* [41], *Mucor hiemalis* [42], *Mucor* sp. [43], *Neosartorya spathulata* [19], *Streptomyces fulvissimus*, *Syncephalastrum racemosum* [21], and *Trichothecium roseum* [44] were reported to perform sulfation of some phenolic compounds. 

Unlike the complicated sulfation procedure of 8-prenylnaringein, which requires a two-step process with the use of a phosphate buffer [21], we obtained the sulfated metabolites of licochalcones and xanthohumol (**7**–**13**) by directly using the cultures of *A. coerulea*. The metabolite **7** was previously identified as a phase II sulfate conjugate of LCA during the metabolism studies using human hepatocytes [5]. Results obtained from this study showed that *A. coerulea* is capable of sulfation of chalcones and exhibits parallels between microbial and mammalian metabolism not only in phase I but also in phase II metabolism [21,31]. It is widely known that the phase II sulfation reactions are mediated by the enzyme sulfotransferases, which catalyze the transfer of sulfonate group from the active sulfate to the substrates containing hydroxyl groups [45]. Thus, the sulfate metabolites of licochalcones and xanthohumol were supposed to be produced by the enzyme sulfotransferase from *A. coeralea*.

To demonstrate the biological activity of compounds **7**, **13** and their aglycones (**1**, **5**), the cytotoxic activities against three different human cancer cell lines were evaluated by using MTT assay (Table 1). Although, as expected, the sulfated metabolites were less active than their aglycones, they still exhibited moderate cytotoxic activities. The glucosylated metabolites displayed weak cytotoxic activity with IC_50_ > 100 μM. From the results obtained (Table 1 and Table S1), it was observed that all of the test compounds, except LCC, exhibited higher cytotoxic activities against A375P cells than MCF-7 and A549 cells. LCC showed stronger activity against MCF-7 cells than A549 cells, which was supported by the result reported by Zheng and colleagues [46]. Zheng and colleagues observed that LCB (**2**) exhibited weak cytotoxic activity against MCF-7 cells with IC_50_ > 80 μM after 24 and 48 h treatment [47], which was similar to our result (Supporting Table S1). All the compounds tested displayed much stronger cytotoxic activities than LCB, indicating that the prenyl group plays a significant role in the cytotoxic activity of these compounds against the tested cancer cell lines. However, Shim and colleagues have reported that LCB exhibited moderate activities against A375 and A431 skin cancer cells (IC_50_ 13.7 and 19.1 μM, respectively) [48], and showed stronger cytotoxic activity against HSC4 oral squamous cell carcinoma cells (IC_50_ 13 μM) than LCA (IC_50_ 20.42 μM), LCC (IC_50_ 27.1 μM), and LCH (IC_50_ 14.4 μM) after 48 h treatment [49,50,51,52]. Though LCA and XN exhibited good activity in most cases, they were found to show low cytotoxic and apoptotic activity against LNCaP prostate cancer cells [53]. Thus, the cytotoxic activity of compounds against different types of cancer cell lines should be evaluated on a case-by-case basis.

## 3. Materials and Methods

### 3.1. Materials and Microorganisms

Licochalcone A (**1**) and demethylzeylasteral (DZ) were purchased from Biopurify Phytochemicals Ltd. (Chengdu, China) and confirmed by ^1^H-NMR. Licochalcones B, C, H and xanthohumol (**2**–**5**) were prepared using previously reported methods [27,28]. All the microorganisms were obtained from the Korean Collection for Type Cultures (KCTC). All of the ingredients for microbial media including dextrose, peptone, malt extract, yeast extract, and potato dextrose broth were purchased from Becton, Dickinson and Company (Sparks, MD, USA). Dulbecco’s Modified Eagle Medium (DMEM) and Antibiotic-Antimycotic were purchased from Gibco (Invitrogen, Carlsbad, CA, USA); fetal bovine serum (FBS) was purchased from Welgene Inc. (Gyeongsan-si, Korea). Phosphate-buffered saline (PBS) tablets were purchased from Takara Korea Biomedical Inc. (Seoul, Korea) and thiazolyl blue tetrazolium bromide (MTT) was from Thermo Fisher Scientific (Waltham, MA, USA).

### 3.2. General Experimental Procedures

The ^1^H- and ^13^C-NMR spectra were obtained in DMSO-*d*_6_ or CD_3_OD on a Bruker Avance III HD 400 spectrometer at 400 and 100 MHz, respectively, using TMS as an internal standard. The chemical shift values (δ) are reported in ppm units, and the coupling constants (J) are in Hz. IR spectra were obtained on a JASCO FT/IR-300E spectrometer (Jasco, Tokyo, Japan). UV spectra were measured with a JASCO V-530 spectrophotometer (Jasco, Tokyo, Japan). HRESIMS was determined on a Synapt G2 mass spectrometer (Waters, Milford, MA, USA). TLC analyses were carried out on precoated silica gel 60 F_254_ plates (Merck, Darmstadt, Germany). The developing system used was chloroform:methanol (5:1, *v/v*) solution, and visualization of the TLC plates was performed using anisaldehyde-H_2_SO_4_ spray reagent. For column chromatography, the adsorbent used was Sephadex LH-20 from GE Healthcare (Chicago, IL, USA). HPLC was performed on a Waters 1525 Binary HPLC Pump (Waters Corp., Milford, MA, USA) connected to a Waters 996 Photodiode Array detector using Zorbax SB-CN (9.4 × 250 mm) with MeOH:H_2_O at a flow rate of 2.0 mL/min.

### 3.3. Screening Procedures

In the screening studies, the actively growing microbial cultures were inoculated in 250 mL Erlenmeyer flasks containing 50 mL of a suitable medium and incubated with gentle agitation (200 rpm) at 25 °C in a temperature-controlled shaking incubator. Then, 100 μL of DMSO solution (20 mg/mL) of substrates **1**–**5** was added to each flask 24 h after inoculation, and further incubated at the same condition for another 7 days. Sampling and TLC monitoring were performed at an interval of 24 h. Culture controls consisted of fermentation cultures in which the microorganisms were grown without the addition of substrates.

### 3.4. Biotransformation of **1** by M. hiemalis KCTC 26779

Scale-up fermentations were carried out under the same temperature-controlled shaking condition with ten 500 mL Erlenmeyer flasks, each containing 125 mL malt medium, and 60 mg licochalcone A dissolved in DMSO was distributed evenly between flasks. After incubation for 4 days, the microbial culture broth was extracted with EtOAc (1.5 L) three times, and the organic layers were combined and concentrated in vacuo. The EtOAc extract (265 mg) was chromatographed by semi-preparative reversed-phase HPLC with 60% MeOH as mobile phase at 2 mL/min to afford metabolites **6** (43 mg).

Licochalcone A 4-O-β-D-glucopyranoside (**6**)

Orange amorphous powder. IR (KBr) ν_max_ cm^−1^: 3387, 2962, 1597, 1506, 1281, 1164, 1031. HRESIMS: *m/z* 523.1943 [M+Na]^+^ (calcd for C_27_H_32_O_9_Na, 523.1944), 501.2120 [M+H]^+^ (calcd for C_27_H_3__3_O_9_, 501.2125).

*Trans* form: UV (MeOH) λ_max_ (log ε) nm: 242 (0.93), 315 (1.23), 372 (1.97). ^1^H-NMR (DMSO-*d*_6_, 400 MHz) δ 8.00 (2H, d, *J* = 8.0 Hz, H-2′/6′), 7.92 (1H, d, *J* = 15.7 Hz, H-β), 7.67 (1H, d, *J* = 15.7 Hz, H-α), 7.59 (1H, s, H-6), 6.90 (2H, d, *J* = 8.0 Hz, H-3′/5′), 6.89 (1H, s, H-3), 6.34 (1H, dd, *J* = 10.7, 17.3 Hz, H-2′′), 5.01 (1H, d, *J* = 7.0 Hz, H-1′′′), 5.01 (1H, dd, *J* = 1.2, 17.3 Hz, H-3′′a), 4.97 (1H, dd, *J* = 1.2, 10.7 Hz, H-3′′b), 3.90 (3H, s, -OCH_3_), 3.77 (1H, m, H-6′′′a), 3.44 (1H, m, H-6′′′b), 3.38 (1H, m, H-2′′′), 3.32 (2H, m, H-3′′′/5′′′), 3.13 (1H, m, H-4′′′), 1.50 (3H, s, H-4′′), 1.49 (3H, s, H-5′′). ^13^C-NMR (DMSO-*d*_6_, 100 MHz) δ 187.7 (C=O), 162.8 (C-4′), 159.4 (C-4), 158.7 (C-2), 148.1 (C-2′′), 138.6 (C-β), 131.4 (C-2′/6′), 129.7 (C-1′), 128.7 (C-5), 127.5 (C-6), 119.7 (C-α), 115.9 (C-1), 115.9 (C-3′/5′), 110.7 (C-3′′), 100.5 (C-1′′′), 100.0 (C-3), 78.0 (C-5′′′), 77.8 (C-3′′′), 73.8 (C-2′′′), 70.7 (C-4′′′), 61.4 (C-6′′′), 56.1 (-OCH3), 40.2 (C-1′′), 28.2 (C-4′′), 27.2 (C-5′′).

*Cis* form: UV (MeOH) λ_max_ (log ε) nm: 260 (1.69), 294 (1.63). ^1^H-NMR (DMSO-*d*_6_, 400 MHz) δ 7.76 (2H, d, *J* = 7.9 Hz, H-2′/6′), 7.09 (1H, s, H-6), 7.03 (1H, d, *J* = 12.8 Hz, H-β), 6.78 (2H, d, *J* = 7.9 Hz, H-3′/5′), 6.76 (1H, s, H-3), 6.44 (1H, d, *J* = 12.8 Hz, H-α), 6.02 (1H, dd, *J* = 10.6, 17.5 Hz, H-2′′), 4.90 (1H, d, *J* = 7.2 Hz, H-1′′′), 4.79 (1H, dd, *J* = 1.2, 17.5 Hz, H-3′′a), 4.76 (1H, dd, *J* = 1.2, 10.6 Hz, H-3′′b), 3.77 (1H, m, H-6′′′a), 3.76 (3H, s, -OCH_3_), 3.44 (1H, m, H-6′′′b), 3.41 (1H, m, H-5′′′), 3.32 (2H, m, H-2′′′/3′′′), 3.13 (1H, m, H-4′′′), 1.24 (3H, s, H-4′′), 1.19 (3H, s, H-5′′). ^13^C-NMR (DMSO-*d*_6_, 100 MHz) δ 193.8 (C=O), 163.0 (C-4′), 157.6 (C-4), 156.7 (C-2), 148.0 (C-2′′), 132.0 (C-β), 131.7 (C-2′/6′), 129.1 (C-6), 128.7 (C-1′), 127.3 (C-5), 125.8 (C-α), 116.5 (C-1), 115.8 (C-3′/5′), 110.3 (C-3′′), 100.5 (C-1′′′), 99.5 (C-3), 77.9 (C-5′′′), 77.8 (C-3′′′), 73.8 (C-2′′′), 70.7 (C-4′′′), 61.4 (C-6′′′), 55.8 (-OCH_3_), 40.0 (C-1′′), 27.6 (C-4′′), 27.0 (C-5′′).

### 3.5. Biotransformation of ***1**–**5*** by A. coerulea KCTC 6936

Scale-up fermentations were carried out under the same temperature-controlled shaking condition with five 500 mL Erlenmeyer flasks, each containing 125 mL suitable media, and 30 mg licochalcone A dissolved in DMSO was distributed evenly between flasks. After incubation for 5 days, the microbial culture broth was extracted with EtOAc (0.75 L) four times, and the organic layers were combined and concentrated in vacuo. The EtOAc extract (396 mg) from *A. coerulea* culture broth was chromatographed by semi-preparative reversed-phase HPLC with 55% MeOH as mobile phase at 2 mL/min to give metabolite **7** (14.86 mg) and a mixture of **7** and **8** (2.55 mg, 1:1.5). A similar scale-up process was performed for licochalcones B (24 mg), C (12 mg), H (24 mg) and xanthohumol (30 mg). The EtOAc extract of LCB (50.84 mg) was chromatographed using Sephadex LH-20 eluted with methanol to give metabolite **9** (1.58 mg) and three fractions, fraction 2 was subjected to reversed-phase HPLC with 70% MeOH as mobile phase at 2 mL/min to afford a mixture of metabolites **9** and **10** (6.85 mg, 3:1). The EtOAc extract of LCC (31.92 mg) was chromatographed using Sephadex LH-20 eluted with methanol to give metabolite **11** (2.23 mg). The EtOAc extract of LCH (50.73 mg) was chromatographed using Sephadex LH-20 eluted with methanol to give metabolite **12** (2.25 mg) and four fractions, fractions 2 and 3 were subjected to reversed-phase HPLC with 70% MeOH as mobile phase at 2 mL/min to afford metabolite **12** (3.80 mg). The EtOAc extract of XN (265 mg) was subjected to reversed-phase HPLC with 70% MeOH as mobile phase at 2 mL/min to afford metabolite **13** (5.2 mg).

#### 3.5.1. Licochalcone A 4′-Sulfate (**7**)

Yellow amorphous powder. IR (neat) ν_max_ cm^−1^: 3503, 2924, 1646, 1599, 1557, 1251, 1212, 1050, 866, 842. UV (MeOH) λ_max_ (log ε) nm: 265 (1.01), 384 (1.78). HRESIMS: *m/z* 417.1024 [M-H]^−^ (calcd for C_21_H_21_O_7_S, 417.1008). ^1^H-NMR (DMSO-*d*_6_, 400 MHz) δ 8.02 (2H, d, *J* = 8.4 Hz, H-2′/6′), 7.92 (1H, d, *J* = 15.6 Hz, H-β), 7.59 (1H, d, *J* = 15.6 Hz, H-α), 7.54 (1H, s, H-6), 7.32 (2H, d, *J* = 8.4 Hz, H-3′/5′), 6.54 (1H, s, H-3), 6.25 (1H, dd, *J* = 10, 17.6 Hz H-2′′), 4.94 (2H, br d, *J* = 14.4 Hz, H-3′′), 3.83 (3H, s, -OCH_3_), 1.45 (6H, s, H-4′′/5′′). ^13^C-NMR (DMSO-*d*_6_, 100 MHz) δ 188.5 (C=O), 160.5 (C-4), 158.9 (C-2), 157.8 (C-4′), 148.0 (C-2′′), 140.0 (C-β), 133.3 (C-1′), 130.1 (C-2′/6′), 128.5 (C-6), 127.1 (C-5), 120.0 (C-3′/5′), 118.3 (C-α), 113.9 (C-1), 110.5 (C-3′′), 100.4(C-3), 56.0 (-OCH_3_), 40.0 (C-1′′), 27.5 (C-4′′/5′′).

#### 3.5.2. Licochalcone A 4-Sulfate (**8**)

Yellow amorphous powder. *Trans* form: UV (MeOH) λ_max_ (log ε) nm: 318 (0.52), 361 (0.64). ^1^H-NMR (CD_3_OD, 400 MHz) δ 8.00 (1H, d, *J* = 15.6 Hz, H-β), 7.96 (2H, d, *J* = 8.4 Hz, H-2′/6′), 7.58 (1H, s, H-6), 7.57 (1H, d, *J* = 15.6 Hz, H-α), 7.51 (1H, s, H-3), 6.89 (2H, d, *J* = 8.4 Hz, H-3′/5′), 6.26 (1H, dd, *J* = 10.5, 17.4 Hz, H-2′′), 5.05 (1H, dd, *J* = 1.3, 17.4 Hz, H-3′′a), 5.04 (1H, dd, *J* = 1.3, 10.5 Hz, H-3′′b), 3.94 (3H, s, -OCH_3_), 1.53 (6H, s, H-4′′/5′′). *Cis* form: UV (MeOH) λmax (log ε) nm: 259 (0.32), 288 (0.27). ^1^H-NMR (CD_3_OD, 400 MHz) δ 7.78 (2H, d, *J* = 8.7 Hz, H-2′/6′), 7.33 (1H, s, H-6), 7.16 (1H, d, *J* = 12.8 Hz, H-β), 7.04 (1H, s, H-3), 6.72 (2H, d, *J* = 8.7 Hz, H-3′/5′), 6.36 (1H, d, *J* = 12.8 Hz, H-α), 5.92 (1H, dd, *J* = 10.7, 17.4 Hz, H-2′′), 4.79 (1H, dd, *J* = 1.4, 10.7 Hz, H-3′′b), 4.76 (1H, dd, *J* = 1.4, 17.4 Hz, H-3′′a), 3.79 (3H, s, -OCH_3_), 1.25 (6H, s, H-4′′/5′′).

#### 3.5.3. Licochalcone B 3-Sulfate (**9**)

Yellow amorphous powder. IR (neat) ν_max_ cm^−1^: 3451, 2929, 1646, 1592, 1258, 1049, 866. UV (MeOH) λ_max_ (log ε) nm: 242 (0.62), 348 (1.46). HRESIMS: *m/z* 365.0366 [M-H]^−^ (calcd for C_16_H_13_O_8_S, 365.0331). ^1^H-NMR (CD_3_OD, 400 MHz) δ 7.99 (1H, d, *J* = 15.6 Hz, H-β), 7.99 (2H, d, *J* = 8.8 Hz, H-2′/6′), 7.66 (1H, d, *J* = 15.6 Hz, H-α), 7.57 (1H, d, *J* = 8.8 Hz, H-6), 6.89 (2H, d, *J* = 8.8 Hz, H-3′/5′), 6.74 (1H, d, *J* = 8.8 Hz, H-5), 4.01 (3H, s, -OCH_3_). ^13^C-NMR (CD_3_OD, 100 MHz) δ 189.8 (C=O), 162.4 (C-4′), 154.1 (C-2), 154.0 (C-4), 139.0 (C-β), 134.0 (C-3), 130.9 (C-2′/6′), 129.8 (C-1′), 125.1 (C-6), 120.7 (C-1), 119.7 (C-α), 115.0 (C-3′/5′), 113.1 (C-5), 61.1 (-OCH_3_). 

#### 3.5.4. Licochalcone B 4-Sulfate (**10**)

UV (MeOH) λ_max_ (log ε) nm: 242 (0.82), 345 (1.99). HRESIMS: *m/z* 365.0336 [M-H]^−^ (calcd for C_16_H_13_O_8_S, 365.0331). ^1^H-NMR (CD_3_OD, 400 MHz) δ 8.02 (1H, d, *J* = 15.7 Hz, H-β), 8.01 (2H, d, *J* = 8.7 Hz, H-2′/6′), 7.73 (1H, d, *J* = 15.7 Hz, H-α), 7.33 (1H, d, *J* = 8.8 Hz, H-6), 7.21 (1H, d, *J* = 8.8 Hz, H-5), 6.90 (2H, d, *J* = 8.7 Hz, H-3′/5′), 3.90 (3H, s, -OCH_3_). ^13^C-NMR (CD_3_OD, 100 MHz) δ 189.6 (C=O), 162.5 (C-4′), 148.7 (C-2), 143.1 (C-4), 143.0 (C-3), 138.4 (C-β), 131.0 (C-2′/6′), 129.6 (C-1′), 125.5 (C-1), 121.7 (C-α), 117.9 (C-5), 117.4 (C-6), 115.1 (C-3′/5′), 61.3 (-OCH_3_). 

#### 3.5.5. Licochalcone C 4-Sulfate (**11**)

Yellow amorphous powder. IR (neat) ν_max_ cm^−1^: 3503, 2925, 1646, 1599, 1557, 1250, 1212, 1050, 866. UV (MeOH) λ_max_ (log ε) nm: 327 (1.82). HRESIMS: *m/z* 417.1010 [M-H]^−^ (calcd for C_21_H_21_O_7_S, 417.1008). *Trans* form: ^1^H-NMR (CD_3_OD, 400 MHz) δ 8.03 (2H, d, *J* = 8.7 Hz, H-2′/6′), 8.01 (1H, d, *J* = 15.9 Hz, H-β), 7.73 (1H, d, *J* = 15.9 Hz, H-α), 7.72 (1H, d, *J* = 8.8 Hz, H-6), 7.43 (1H, d, *J* = 8.7 Hz, H-5), 6.90 (2H, d, *J* = 8.7 Hz, H-3′/5′), 5.26 (1H, t, *J* = 7.1 Hz, H-2′′), 3.77 (3H, s, -OCH_3_), 3.49 (2H, d, *J* = 7.1 Hz, H-1′′), 1.81 (3H, s, H-4′′), 1.67 (3H, s, H-5′′). ^13^C-NMR (CD_3_OD, 100 MHz) δ 189.6 (C=O), 162.5 (C-4′), 159.0 (C-2), 150.5 (C-4), 138.6 (C-β), 131.0 (C-2′/6′), 130.8 (C-3′′), 129.6 (C-1′), 128.8 (C-3), 125.4 (C-6), 124.7 (C-1), 122.6 (C-2′′), 121.5 (C-α), 117.4 (C-5), 115.1 (C-3′/5′), 61.8 (-OCH_3_), 24.5 (C-4′′), 23.2 (C-1′′), 16.7 (C-5′′). 

*Cis* form: ^1^H-NMR (CD_3_OD, 400 MHz) δ 7.84 (2H, d, *J* = 8.8 Hz, H-2′/6′), 7.13 (1H, d, *J* = 8.7 Hz, H-5), 7.10 (1H, d, *J* = 12.9 Hz, H-β), 7.07 (1H, d, *J* = 8.7 Hz, H-6), 6.76 (2H, d, *J* = 8.8 Hz, H-3′/5′), 6.65 (1H, d, *J* = 12.9 Hz, H-α), 5.14 (1H, t, *J* = 6.8 Hz, H-2′′), 3.71 (3H, s, -OCH_3_), 3.39 (2H, d, *J* = 6.8 Hz, H-1′′), 1.76 (3H, s, H-4′′), 1.65 (3H, s, H-5′′). ^13^C-NMR (CD_3_OD, 100 MHz) δ 194.4 (C=O), 162.6 (C-4′), 157.4 (C-2), 151.9 (C-4), 133.5 (C-β), 131.3 (C-2′/6′), 130.5 (C-3′′), 128.3 (C-1′), 127.8 (C-3), 127.4 (C-6), 126.9 (C-α), 125.7 (C-1), 122.8 (C-2′′), 116.5 (C-5), 114.9 (C-3′/5′), 60.8 (-OCH_3_), 24.5 (C-4′′), 23.1 (C-1′′), 16.7 (C-5′′). 

#### 3.5.6. Licochalcone H 4-Sulfate (**12**)

Yellow amorphous powder. IR (neat) ν_max_ cm^−1^: 3436, 2959, 2925, 1646, 1599, 1557, 1249, 1212, 1050, 866. UV (MeOH) λ_max_ (log ε) nm: 306 (1.87), 359 (1.54). HRESIMS: *m/z* 417.0974 [M-H]^−^ (calcd for C_21_H_21_O_7_S, 417.1008). *Trans* form ^1^H-NMR (CD_3_OD, 400 MHz) δ 7.98 (1H, d, *J* = 15.8 Hz, H-β), 7.97 (2H, d, *J* = 8.9 Hz, H-2′/6′), 7.68 (1H, d, *J* = 15.8 Hz, H-α), 7.49 (1H, s, H-6), 7.26 (1H, s, H-3), 6.89 (2H, d, *J* = 8.9 Hz, H-3′/5′), 5.35 (1H, t, *J* = 7.4 Hz, H-2′′), 3.93 (3H, s, -OCH_3_), 3.42 (2H, d, *J* = 7.4 Hz, H-1′′), 1.76 (3H, s, H-4′′), 1.75 (3H, s, H-5′′). ^13^C-NMR (CD_3_OD, 100 MHz) δ 191.8 (C=O), 163.7 (C-4′), 159.2 (C-2), 154.6 (C-4), 140.8 (C-β), 134.4 (C-3′′), 132.3 (C-2′/6′), 131.2 (C-6), 130.7 (C-1′), 126.9 (C-5), 123.8 (C-2′′), 122.2 (C-α), 121.3 (C-1), 116.4 (C-3′/5′), 106.1 (C-3), 56.4 (-OCH_3_), 28.8 (C-1′′), 25.9 (C-4′′), 18.0 (C-5′′). 

*Cis* form ^1^H-NMR (CD_3_OD, 400 MHz) δ 7.81 (2H, d, *J* = 8.9 Hz, H-2′/6′), 7.12 (1H, d, *J* = 12.8 Hz, H-β), 7.06 (1H, s, H-3), 6.93 (1H, s, H-6), 6.76 (2H, d, *J* = 8.9 Hz, H-3′/5′), 6.43 (1H, d, *J* = 12.8 Hz, H-α), 5.01 (1H, t, *J* = 7.4 Hz, H-2′′), 3.76 (3H, s, -OCH_3_), 3.17 (2H, d, *J* = 7.4 Hz, H-1′′), 1.63 (3H, s, H-4′′), 1.55 (3H, s, H-5′′). ^13^C-NMR (CD_3_OD, 100 MHz) δ 197.1 (C=O), 164.0 (C-4′), 157.1 (C-2), 152.8 (C-4), 133.9 (C-β), 133.5 (C-3′′), 132.9 (C-2′/6′), 131.9 (C-6), 129.9 (C-1′), 127.4 (C-α), 126.6 (C-5), 123.4 (C-2′′), 122.2 (C-1), 116.2 (C-3′/5′), 105.6 (C-3), 56.0 (-OCH_3_), 28.1 (C-1′′), 25.9 (C-4′′), 17.7 (C-5′′). 

#### 3.5.7. Xanthohumol 4′-Sulfate (**13**)

Yellow amorphous powder. IR (neat) ν_max_ cm^−1^: 3447, 2924, 1645, 1598, 1559, 1241, 1213, 1051, 867. UV (MeOH) λ_max_ (log ε) nm: 297 (1.01, sh), 362 (2.18). HRESIMS: *m/z* 433.1092 [M-H]^−^ (calcd. for C_21_H_21_O_8_S, 433.0957). ^1^H-NMR (CD_3_OD, 400 MHz) δ 7.79 (1H, d, *J* = 15.8 Hz, H-β), 7.72 (1H, d, *J* = 15.8 Hz, H-α), 7.52 (2H, d, *J* = 8.5 Hz, H-2/6), 6.89 (1H, s, H-5′), 6.83 (2H, d, *J* = 8.5 Hz, H-3/5), 5.23 (1H, t, *J* = 7.3 Hz, H-2′′), 3.95 (3H, s, -OCH_3_), 3.36 (2H, d, *J* = 7.4 Hz, H-1′′), 1.79 (3H, s, H-4′′), 1.65 (3H, s, H-5′′). ^13^C-NMR (CD_3_OD, 100 MHz) δ 193.8 (C=O), 163.4 (C-2′), 159.9 (C-6′), 159.8 (C-4), 156.5 (C-4′), 143.0 (C-β), 130.4 (C-3′′), 130.1 (C-2/6), 126.8 (C-1′), 124.2 (C-α), 122.2 (C-2′′), 115.5 (C-3/5), 113.9 (C-3′), 108.3 (C-1′), 94.9 (C-5′), 55.1 (-OCH_3_), 24.6 (C-4′′), 21.7 (C-1′′), 16.7 (C-5′′). 

### 3.6. Cytotoxic Activity Evaluation

The cytotoxic activities of the compounds LCA, XN, and their metabolites against A375P (human melanoma), MCF-7 (human breast adenocarcinoma), and A549 (human lung carcinoma) cancer cell lines were performed by using MTT assay, which is based on the reduction of MTT to formazan by mitochondrial dehydrogenase. Briefly, the cells (5~6 × 10^3^/100 μL medium) were treated with each compound and incubated for 24 h (A375P, MCF-7) and 48 h (A549) at 37 °C. After the MTT reagent (0.5 mg/mL) was added to the well and incubated for another 4 h, the supernatant was then removed and the MTT-formazan was dissolved in 100 μL DMSO. The amount of formed formazan was quantified by measuring the absorbance at 490 nm using a microplate reader. Demethylzeylasteral (DZ) was used as a positive control and DMSO was used as a negative control in all experiments.

### 3.7. Hydrolysis of Compounds ***6**–**13***

A solution of compound **6** in 1 N HCl was heated for 2 h. After cooling, the reaction mixture was neutralized and partitioned between EtOAc and H_2_O. The EtOAc and H_2_O extracts were analyzed by TLC, respectively. By comparing the TLC with those of compound **1** and D-glucose, the agylcone of **6** was found to be that of **1** and the monosaccharide was confirmed to be D-glucose [29].

Compounds **7**–**13** were dissolved in MeOH and mixed with 3% HCl at room temperature, respectively. Then MeOH was evaporated, and the aglycone extracted with EtOAc. The residue was analyzed by TLC and HPLC by direct comparison with substrates **1**–**5**. When BaCl_2_ was added to the aqueous layer, a white precipitate was formed which proved the presence of BaSO_4_ [31].

## 4. Conclusions

In the present study, nine conjugated metabolites of licochalcones and xanthohumol were obtained by using the fungi *M. hiemalis* and *A. coerulea*. Among them, the glucosylated metabolite of LCA (**6**), and the sulfated metabolites of LCB, LCC, LCH (**9**–**12**) have never been reported. The glucoside and sulfate moieties substituted at C4-OH were thought to be exhibited as the *cis*-*trans* isomerization barriers, which led to the metabolites (**6**, **11** and **12**) existing as a mixture of *trans-* and *cis*-isomers with the *cis* form as major ones. By using the fungus *A. coerulea*, it was possible to obtain the sulfated compounds of licochalcones A-C, H and xanthohumol. The sulfation reaction is one of the major phase II metabolic pathways in mammalian metabolism of xenobiotics, thus these sulfated metabolites are of significant interest, for they can be used as the reference standards for the detection and identification of the metabolic products of **1**–**5** that occur in mammalian systems. Comparison studies of the cytotoxic activities of the parent compounds and their sulfated metabolites against human cancer cell lines showed that sulfation of LCA and XN reduced but did not inactivate the cytotoxic activity.

## Figures and Tables

**Table 1:** Cytotoxic activities of compounds against cancer cell lines.

Compound	Cell Lines (IC_50_, μM)		
	A375P	MCF-7	A549
**1**	12.86 ± 3.42	19.16 ± 0.65	18.14 ± 1.26
**5**	11.02 ± 2.09	23.11 ± 1.36	26.93 ± 1.56
**7**	27.35 ± 1.44	30.85 ± 4.58	43.07 ± 1.63
**13**	41.04 ± 1.61	81.27 ± 3.05	99.40 ± 1.99
DZ ^1^	9.86 ± 0.57	5.12 ± 0.44	4.01 ± 0.78

^1^ Used as positive control.

## Data Availability

Not applicable.

## Supplementary Materials

The following are available online at https://www.mdpi.com/article/10.3390/ijms22136893/s1, Figures S1–S29: 1D, 2D NMR and HRESIMS spectra of **6**–**13**.
