# Supplementary material for: Microbial Conjugation Studies of Licochalcones and Xanthohumol
Source: Int J Mol Sci. 2021 Jun 26;22(13):6893. doi: 10.3390/ijms22136893 (PMC8268106; doi:10.3390/ijms22136893)
Supplement: Supplementary file 1 [file ijms-22-06893-s001.zip › ijms-1256271-supplementary.pdf]

Supporting material

Microbial Conjugation Studies of Licochalcones and Xanthohumol

Fubo Han, Yina Xiao and Ik-Soo Lee *

College of Pharmacy, Chonnam National University, Gwangju 61186, Republic of Korea; hanfubo0306@gmail.com (F.H.); yogurtxiao@163.com (Y.X)

* Correspondence: islee@chonnam.ac.kr ; Tel.: +82-62-530-2932

Table S1. Cytotoxic activities of compounds **2-4** against cancer cell lines.

Compound	Cell Lines (IC_{50}, μM)		
	A375P	MCF-7	A549
**2**	>100	>100	>100
**3**	19.34±2.47	13.90±0.30	35.27±6.56
**4**	24.17±0.37	36.99±2.49	34.42±4.88

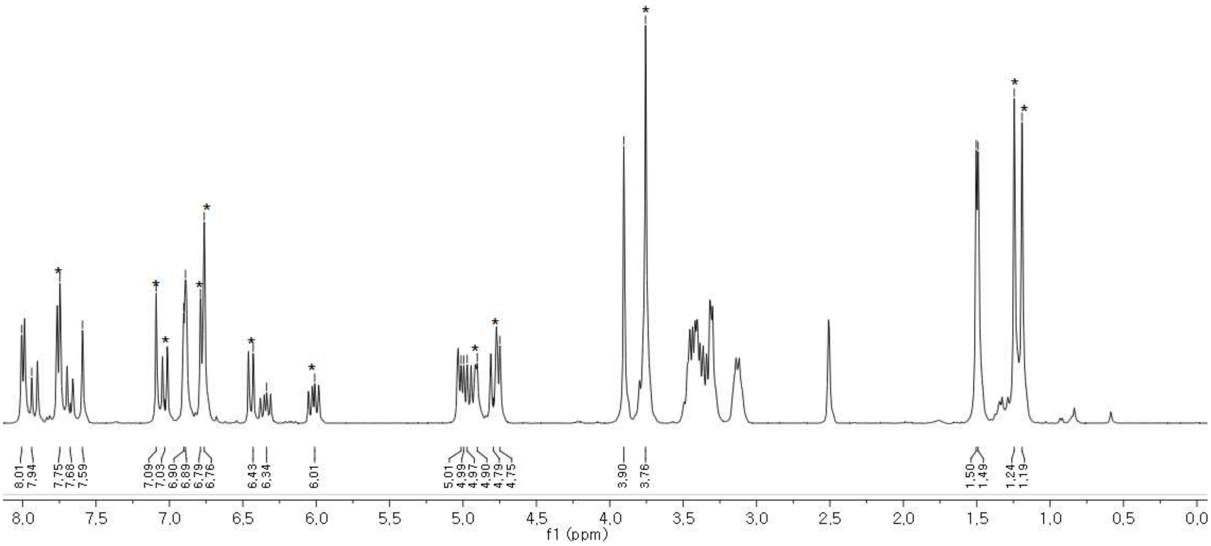

Figure S1. ^{1}H-NMR Spectrum of metabolite **6** (400 MHz, in DMSO-d_6).

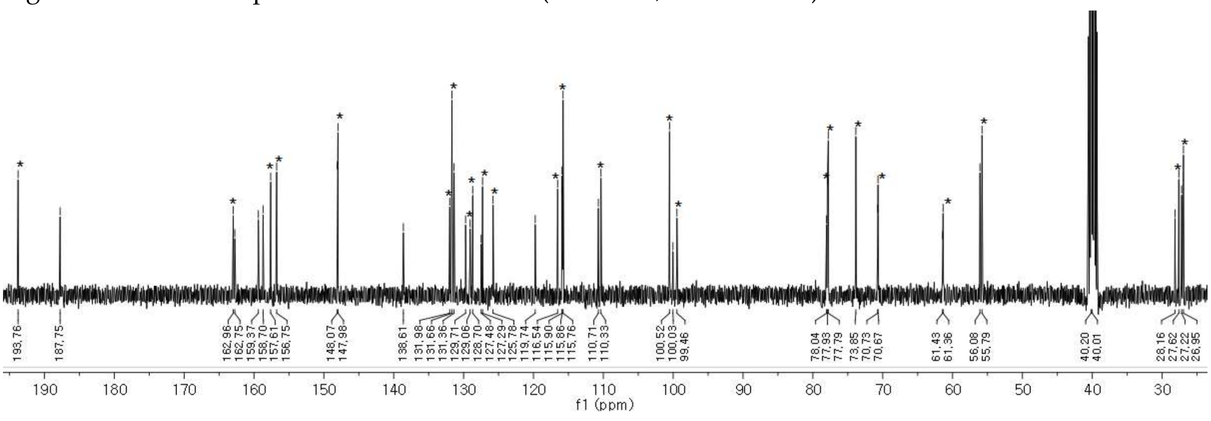

Figure S2. ^{13}C-NMR Spectrum of metabolite **6** (100 MHz, in DMSO-d_6).

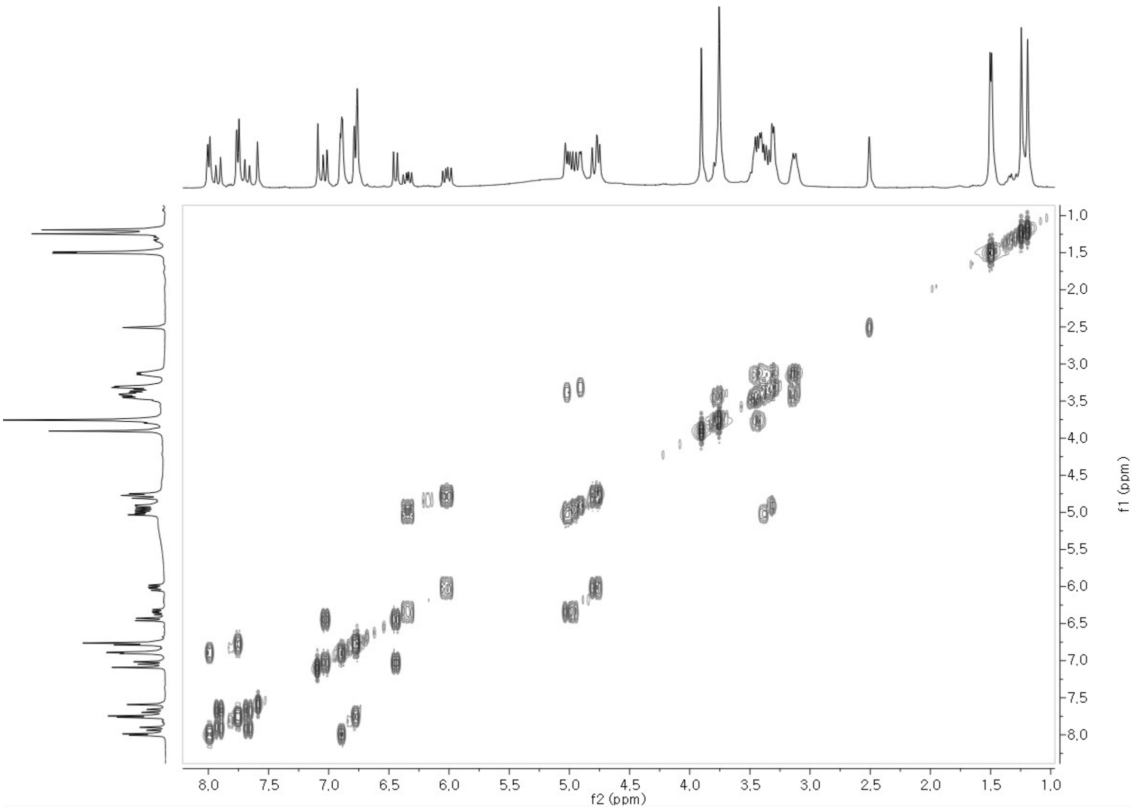

Figure S3. COSY spectrum of metabolite **6** (400 MHz, in DMSO-d_6).

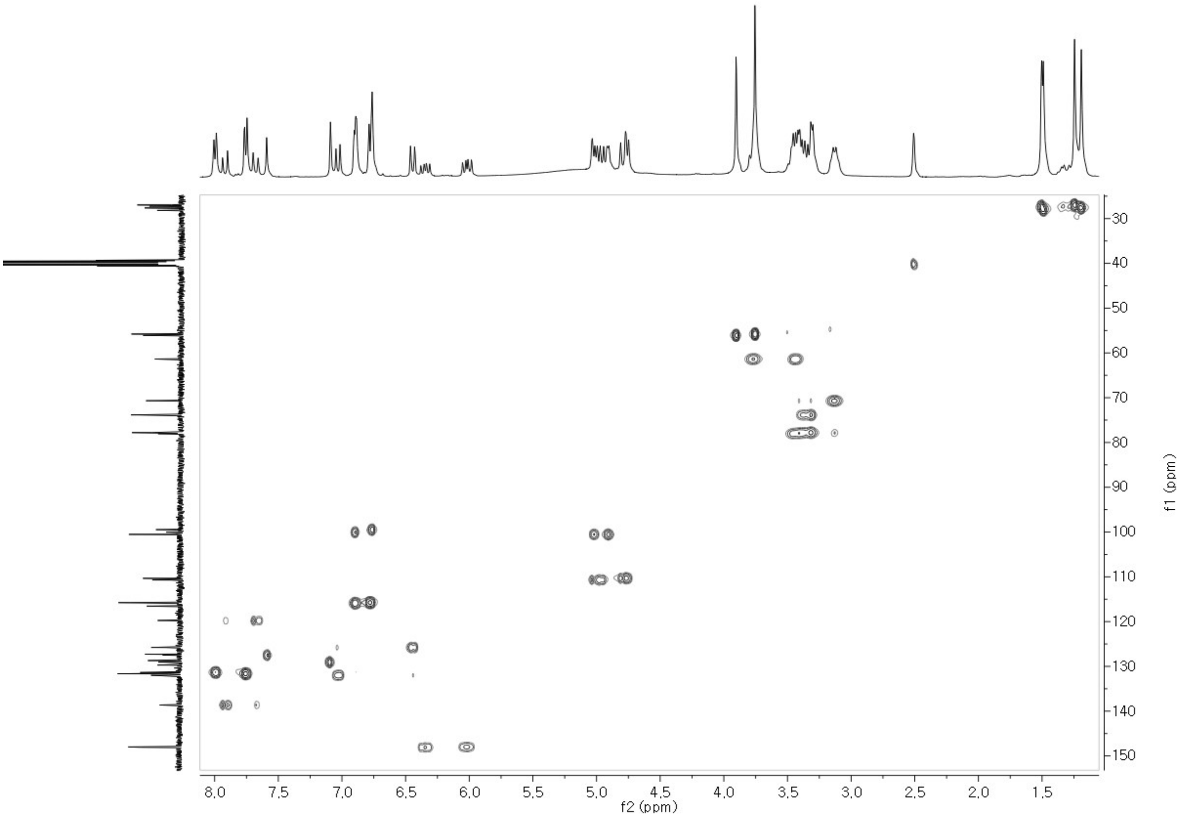

Figure S4. HSQC spectrum of metabolite **6** (400 MHz, in DMSO-d_6).

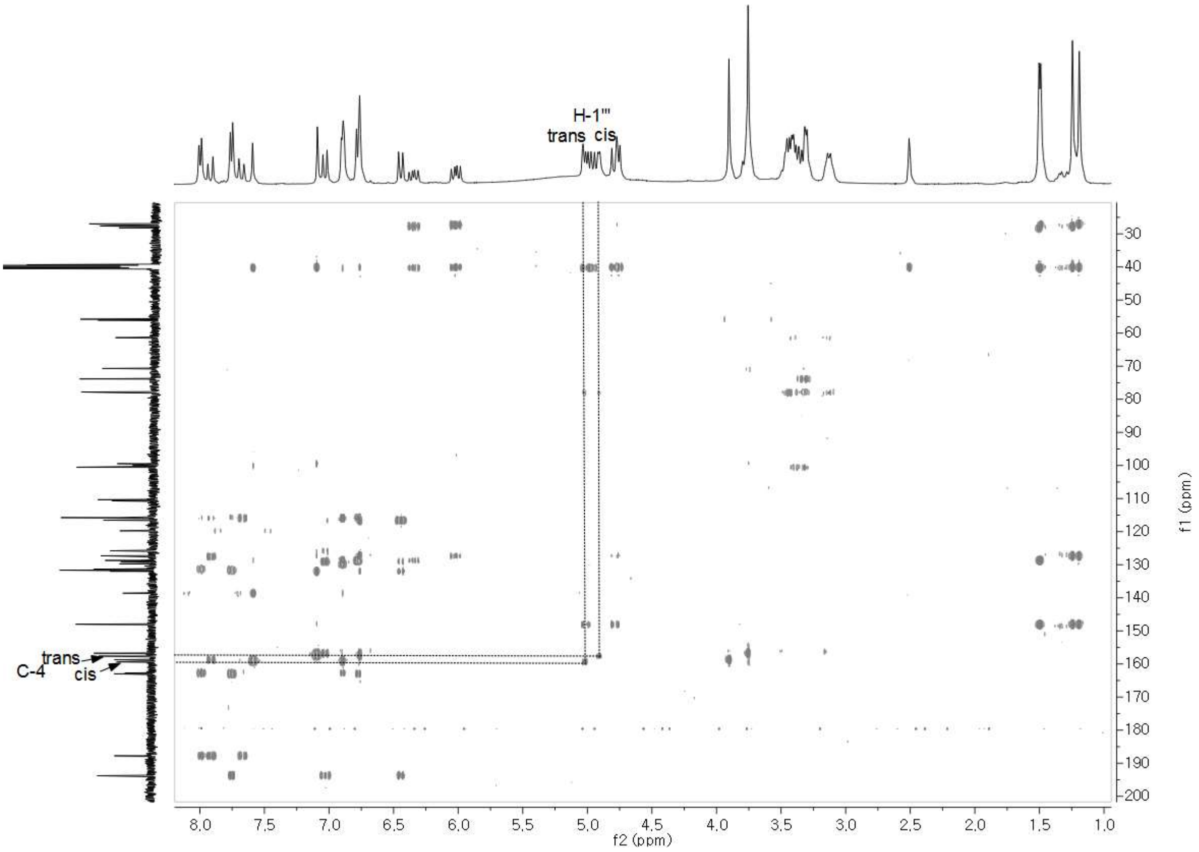

Figure S5. HMBC spectrum of metabolite **6** (400 MHz, in DMSO-d_6).

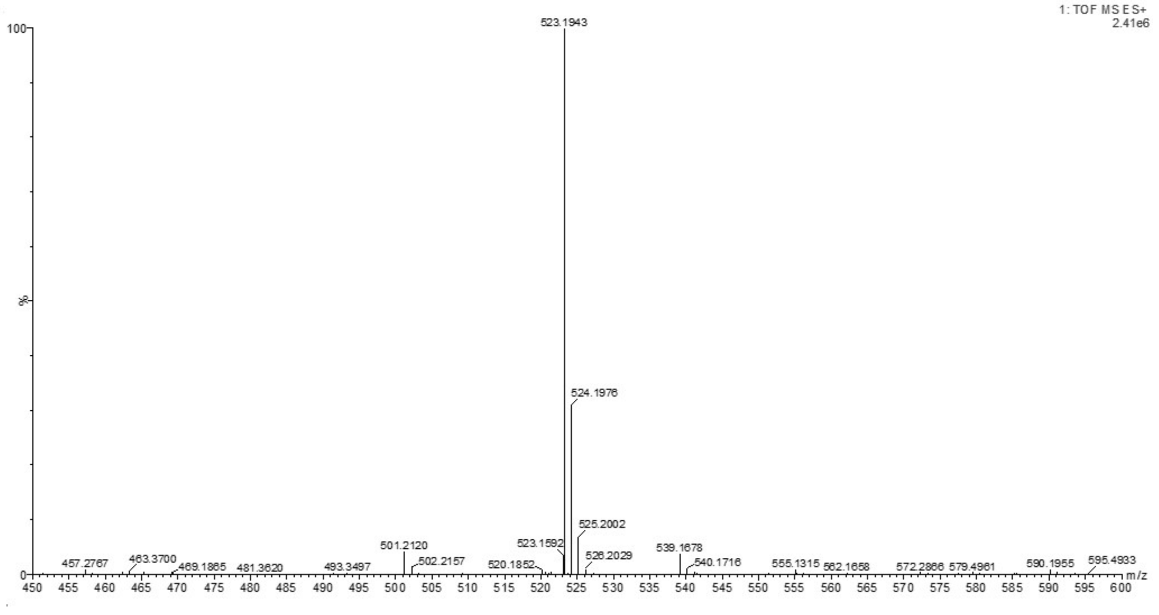

Figure S6. HRESIMS spectrum of metabolite **6**.

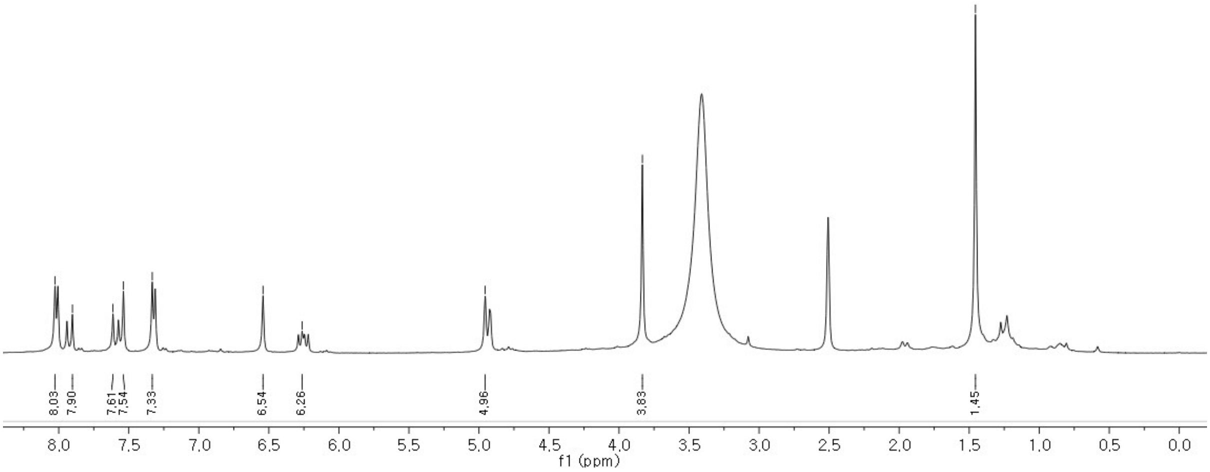

Figure S7. ^{1}H-NMR Spectrum of metabolite **7** (400 MHz, in DMSO-d_6).

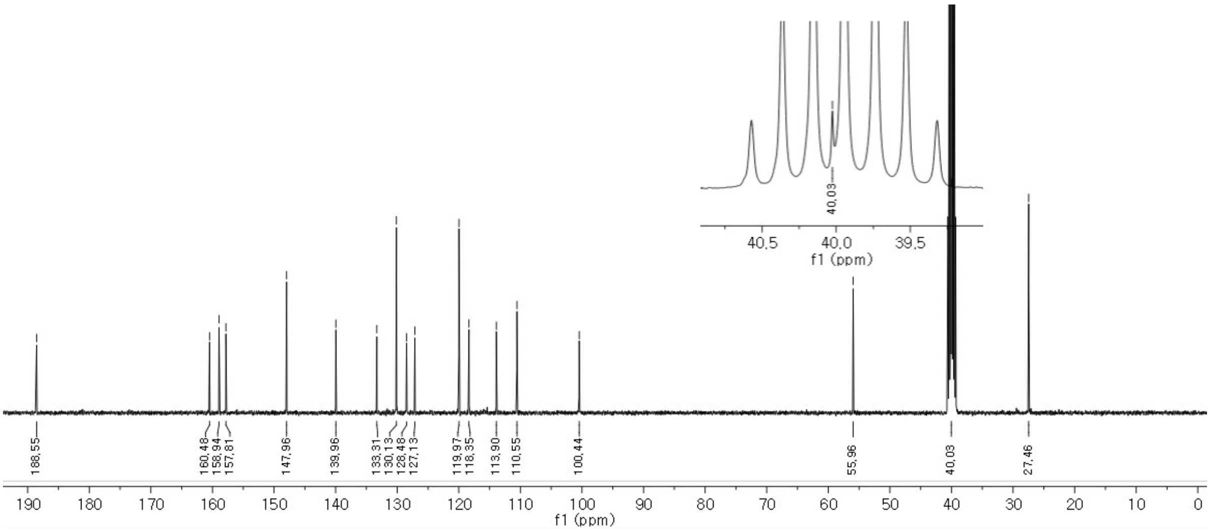

Figure S8. ^{13}C-NMR Spectrum of metabolite **7** (100 MHz, in DMSO-d_6).

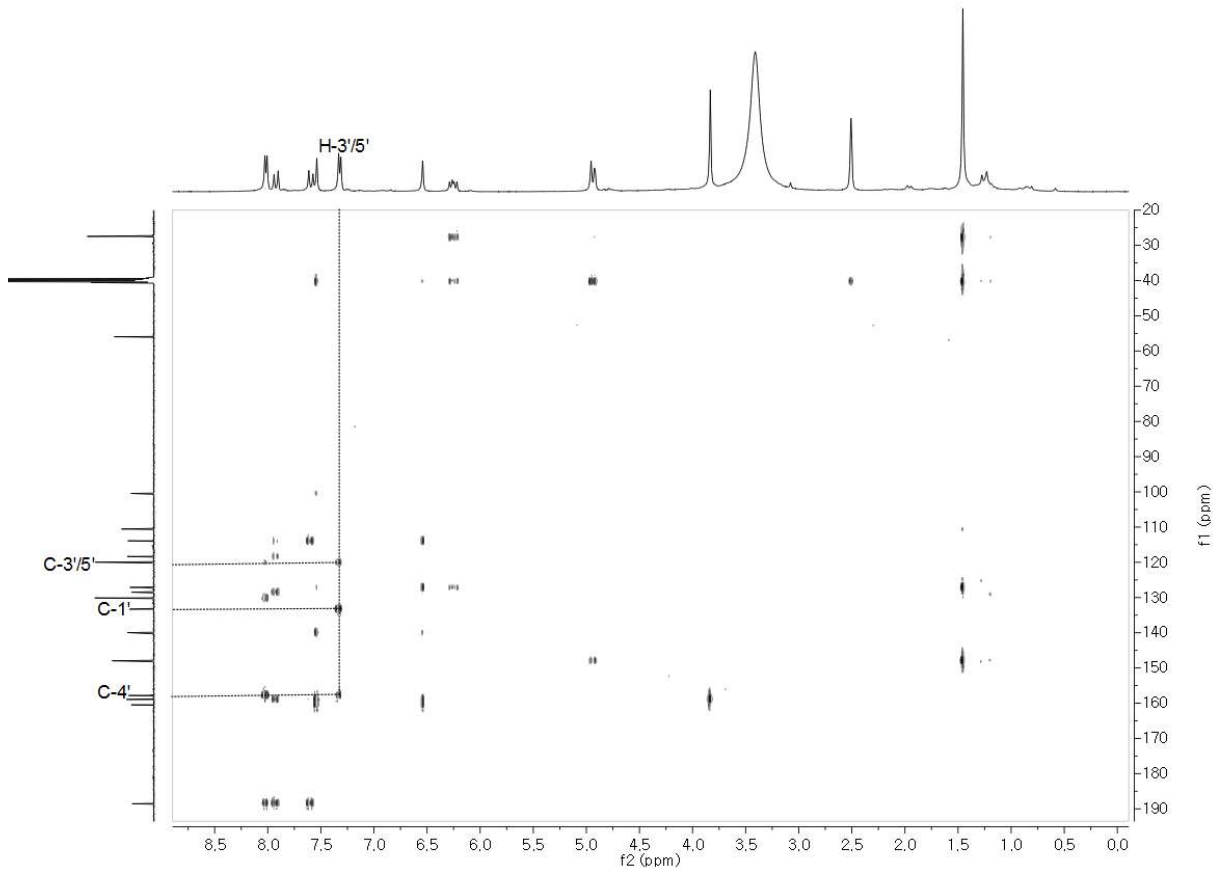

Figure S9. HMBC Spectrum of metabolite **7** (400 MHz, in DMSO-d_6).

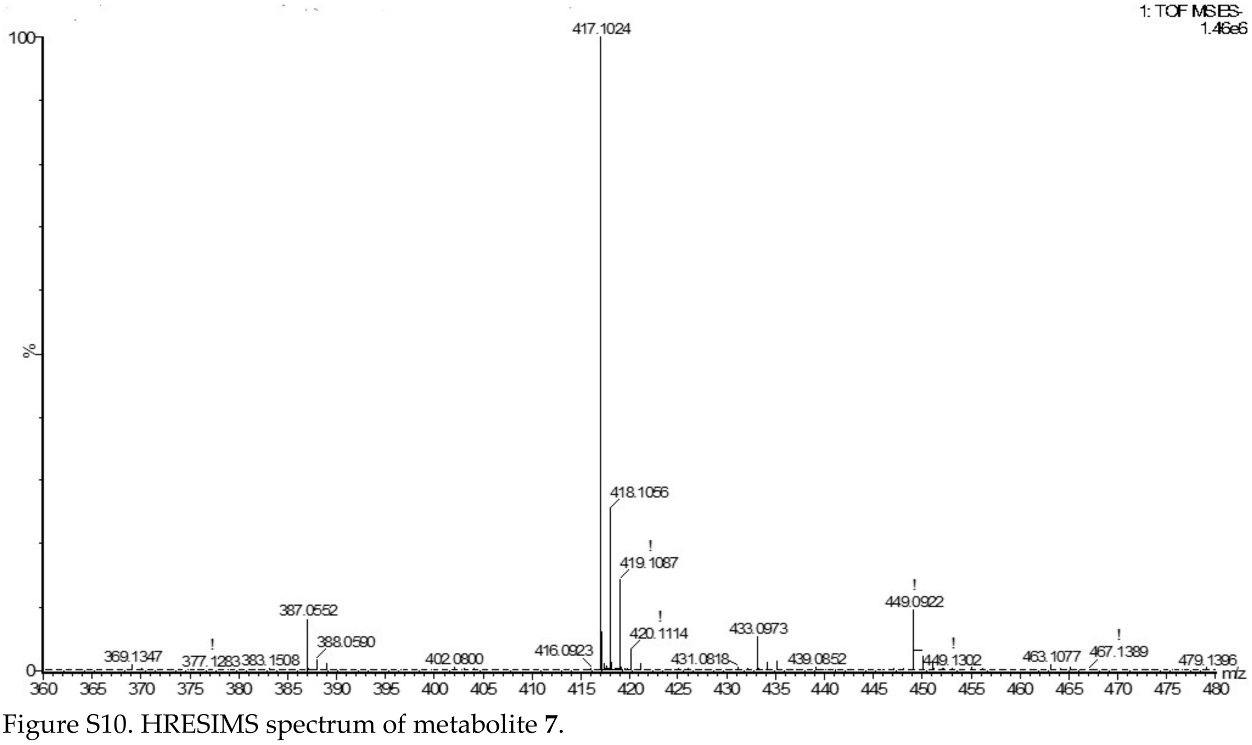

Figure S10. HRESIMS spectrum of metabolite **7**.

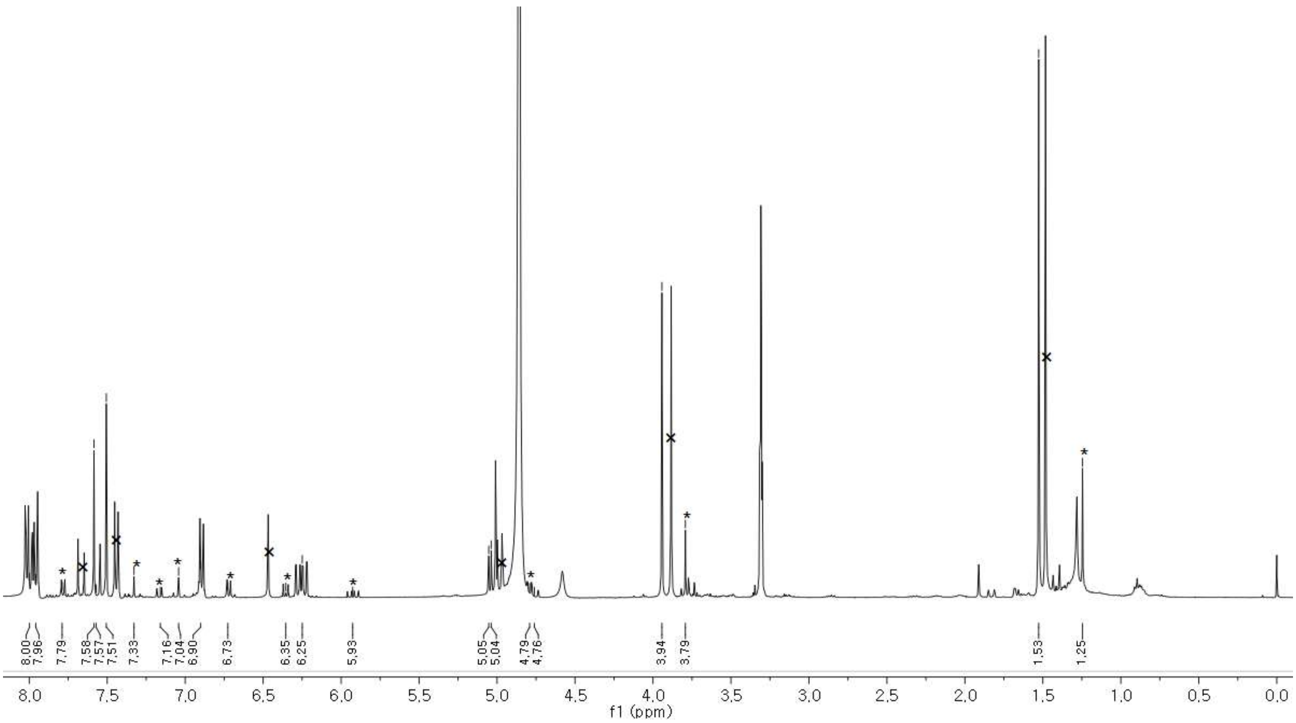

Figure S11. ^{1}H NMR spectrum of metabolite **8** (400 MHz, in CD_3OD). * Signals of *cis* form.

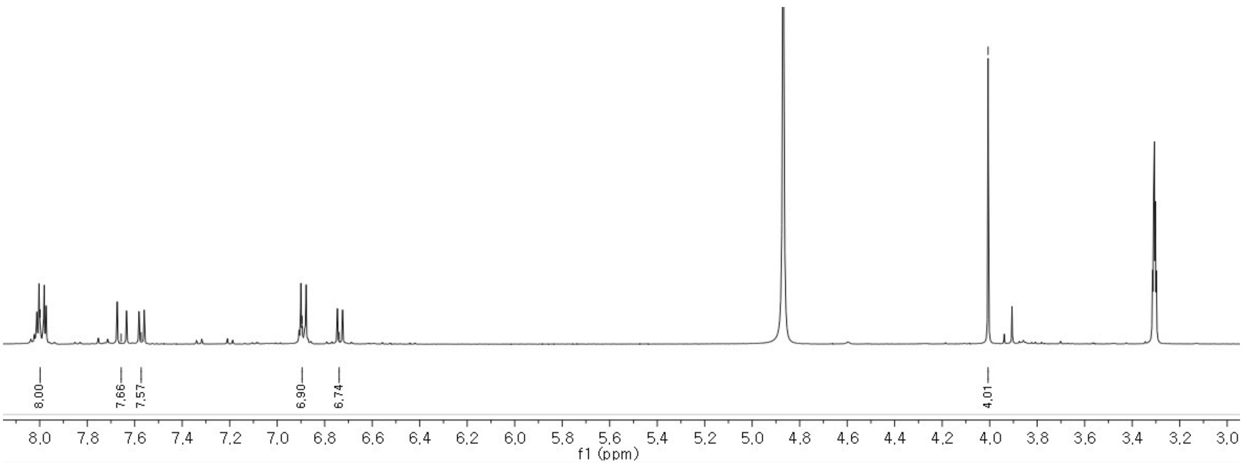

Figure S12. ^{1}H NMR spectrum of metabolite **9** (400 MHz, in CD_3OD).

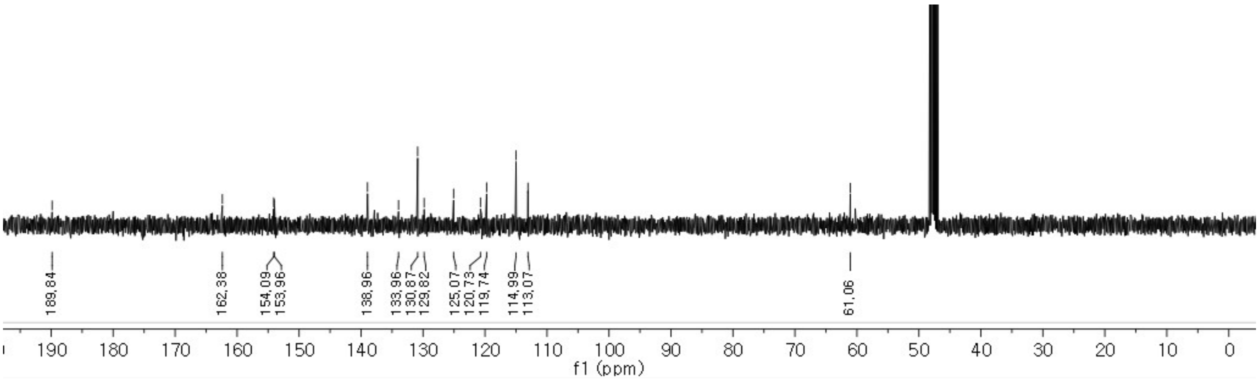

Figure S13. ^{13}C NMR spectrum of metabolite **9** (100 MHz, in CD_3OD).

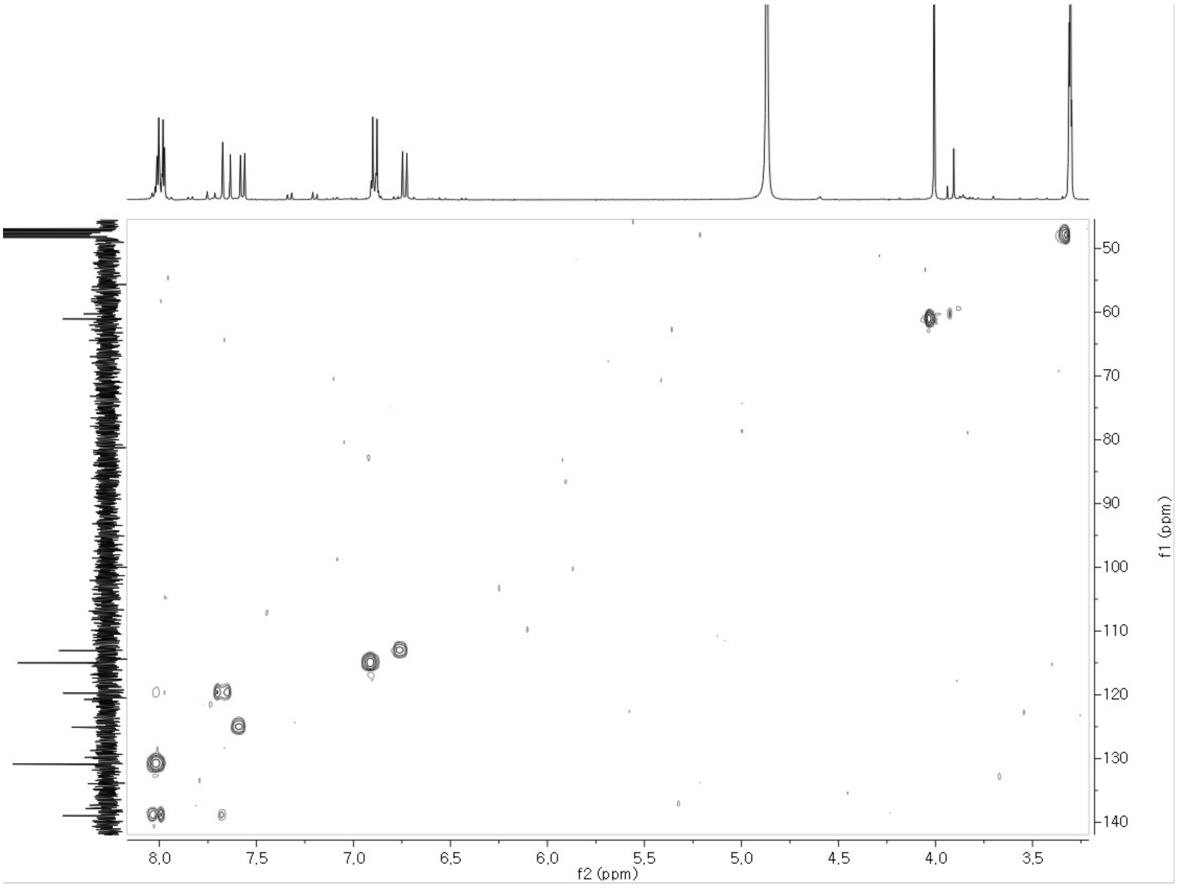

Figure S14. HSQC spectrum of metabolite **9** (400 MHz, in CD_3OD).

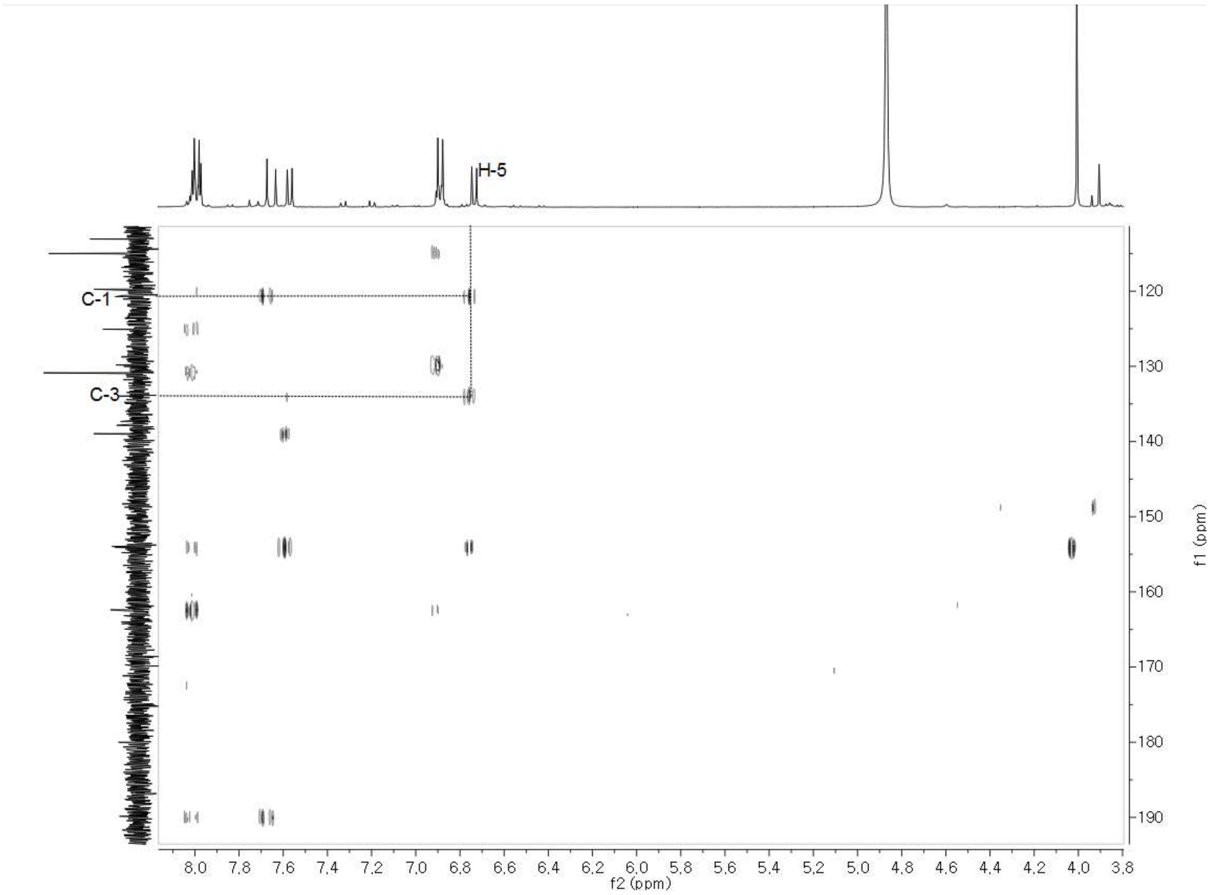

Figure S15. HMBC spectrum of metabolite **9** (400 MHz, in CD_3OD).

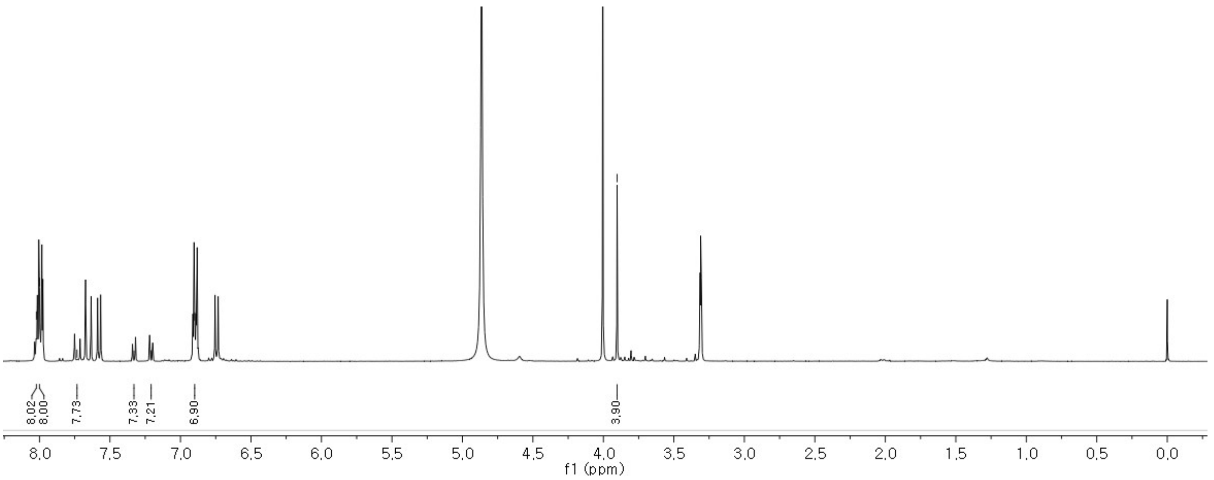

Figure S16. ^{1}H NMR spectrum of metabolite **10** (400 MHz, in CD_3OD).

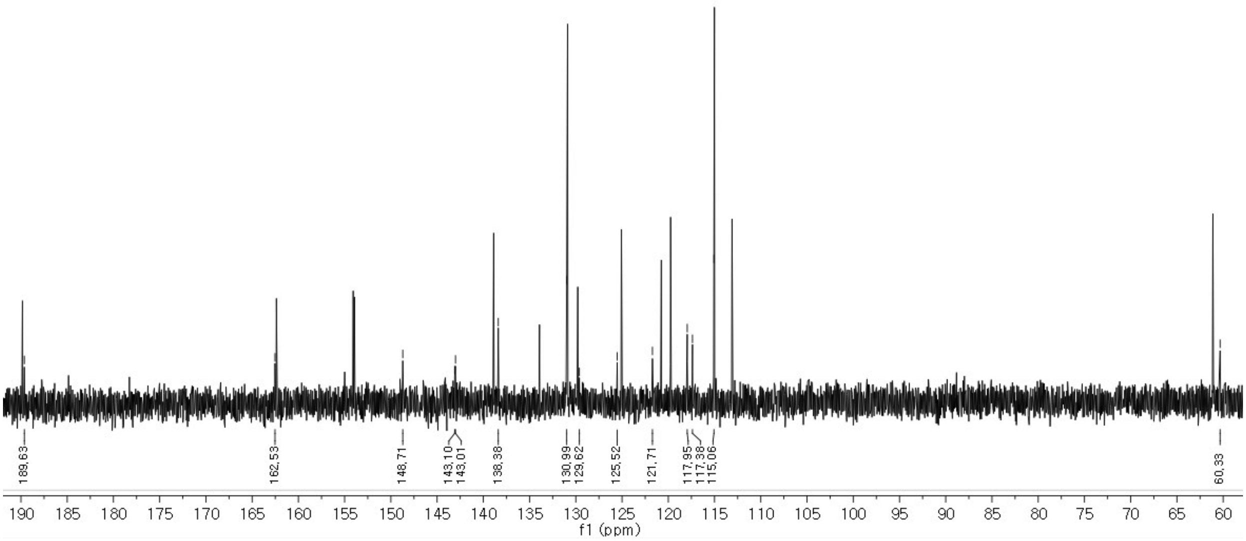

Figure S17. ^{13}C NMR spectrum of metabolite **10** (100 MHz, in CD_3OD).

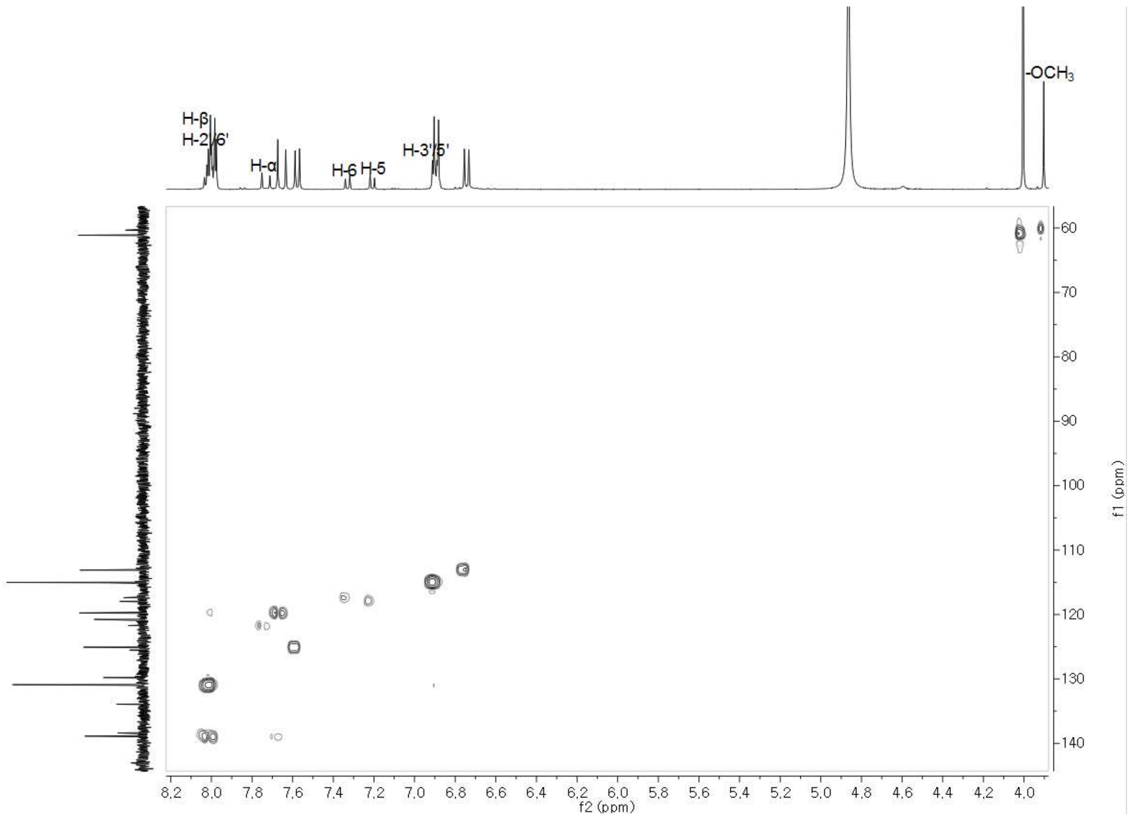

Figure S18. HSQC spectrum of metabolite **10** (400 MHz, in CD_3OD).

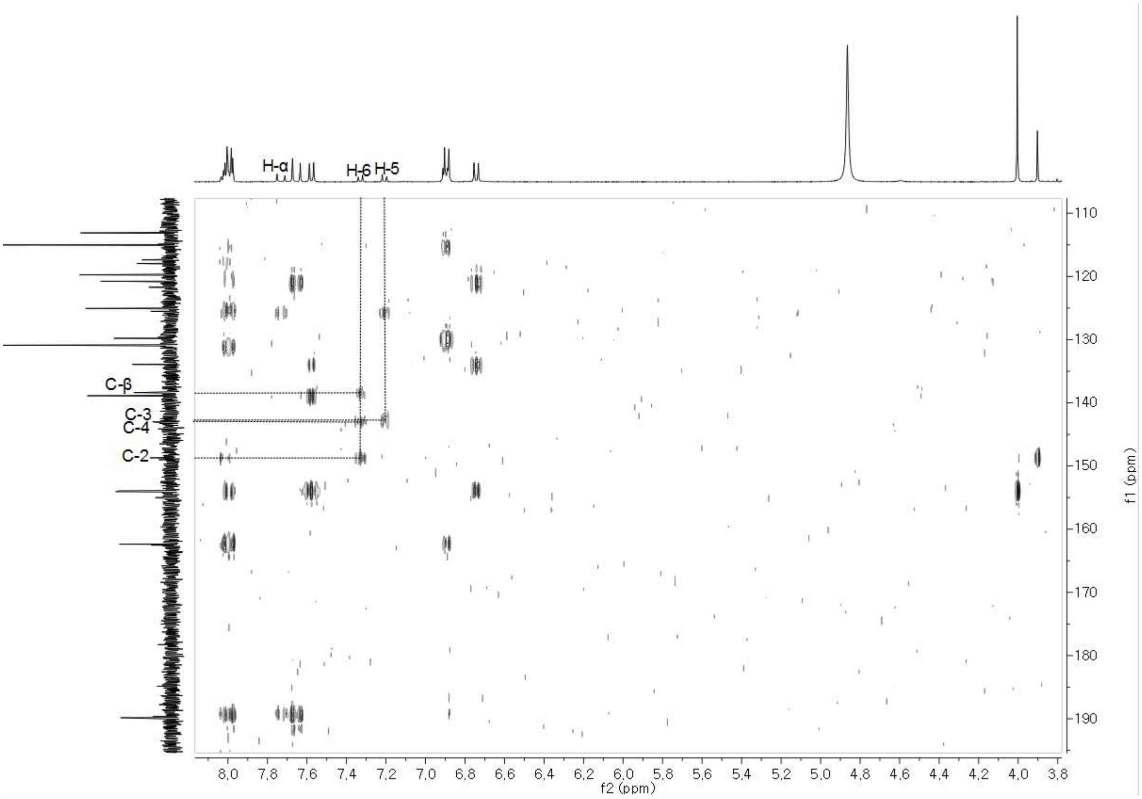

Figure S19. HMBC spectrum of metabolite **10** (400 MHz, in CD_3OD).

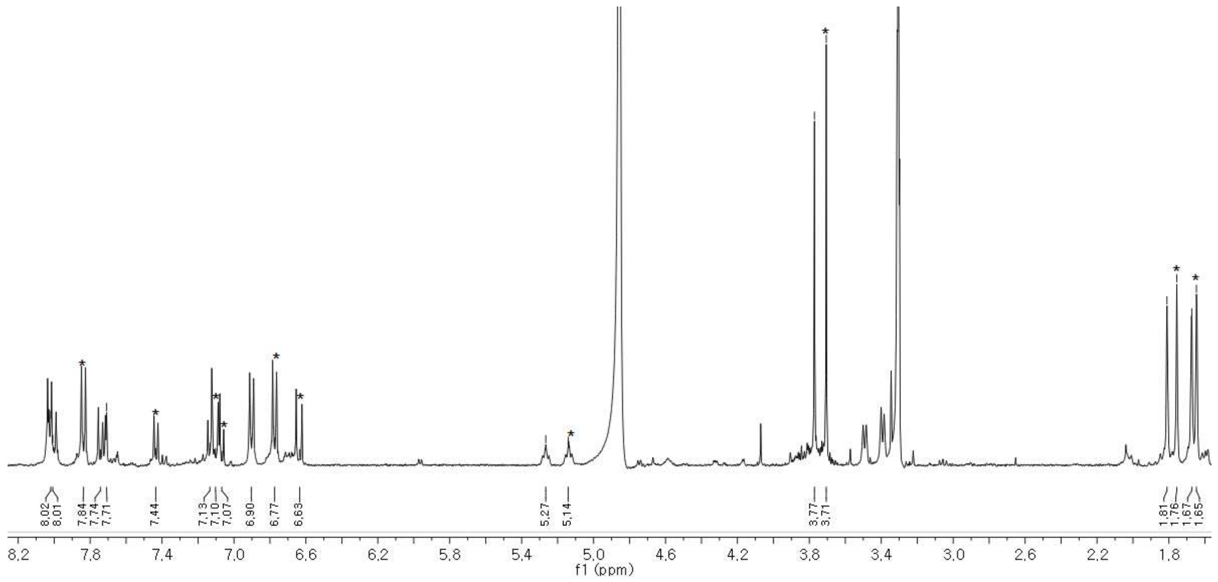

Figure S20. ^{1}H NMR spectrum of metabolite **11** (400 MHz, in CD_3OD). * Signals of *cis* form.

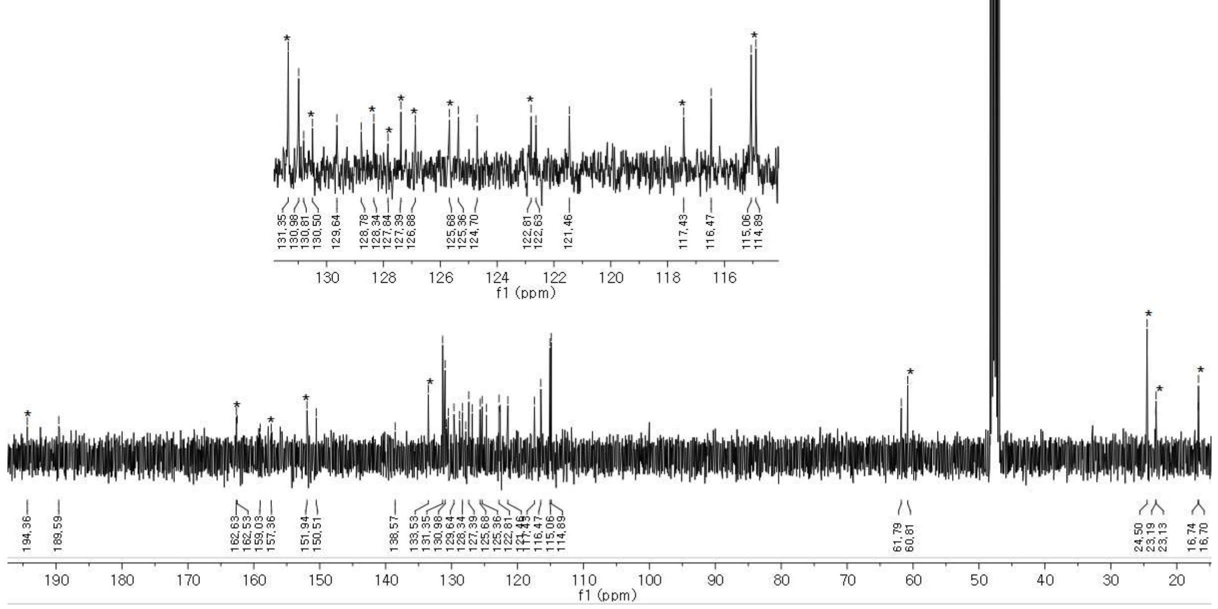

Figure S21. ^{13}C NMR spectrum of metabolite **11** (100 MHz, in CD_3OD). * Signals of *cis* form.

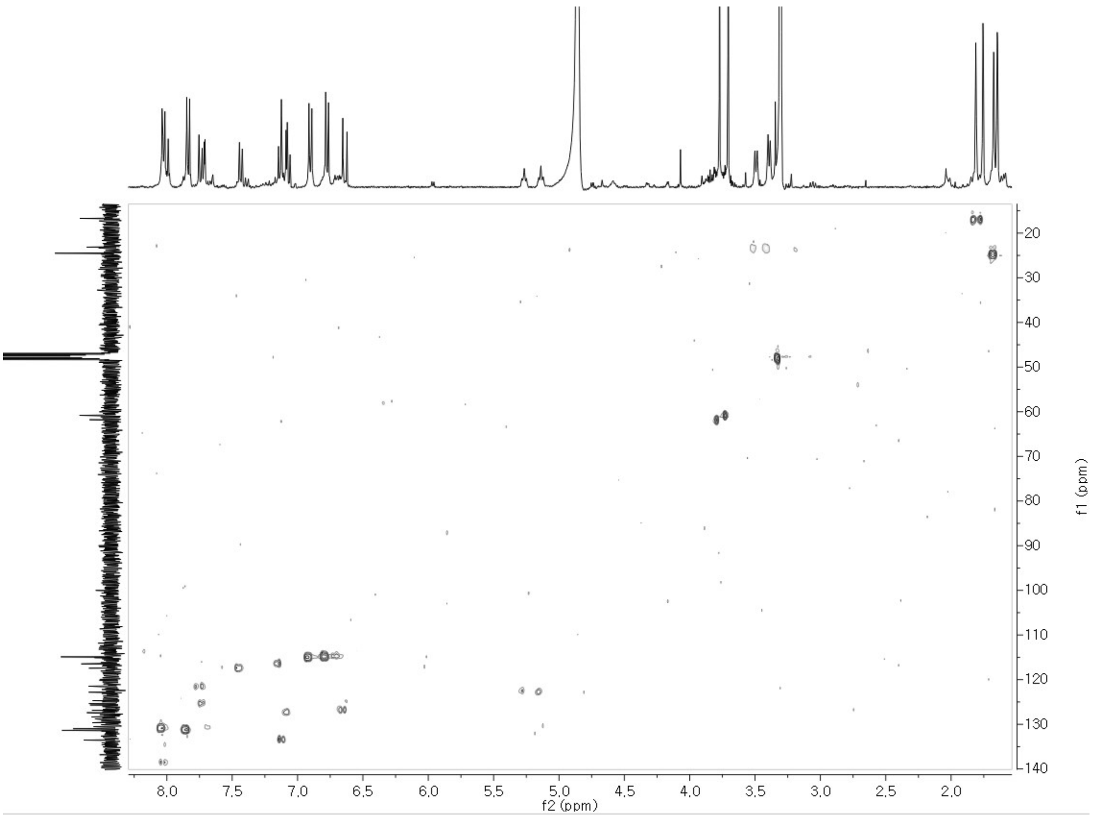

Figure S22. HSQC spectrum of metabolite **11** (400 MHz, in CD_3OD).

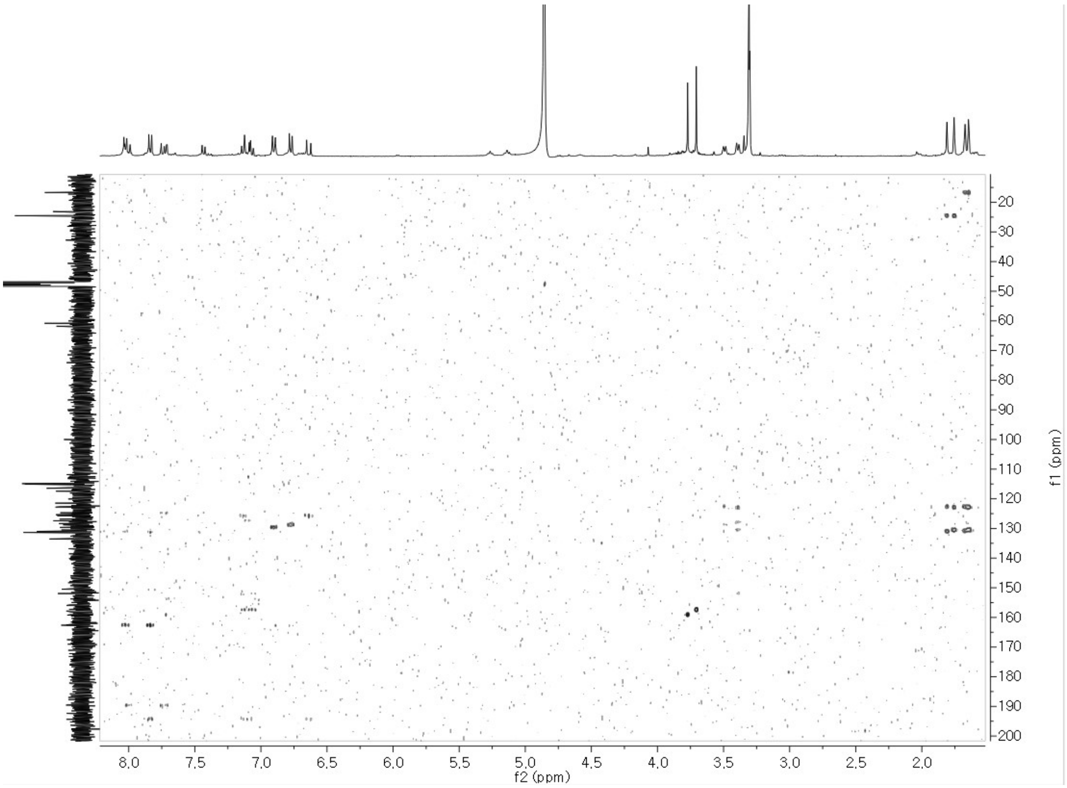

Figure S23. HMBC spectrum of metabolite **11** (400 MHz, in CD_3OD).

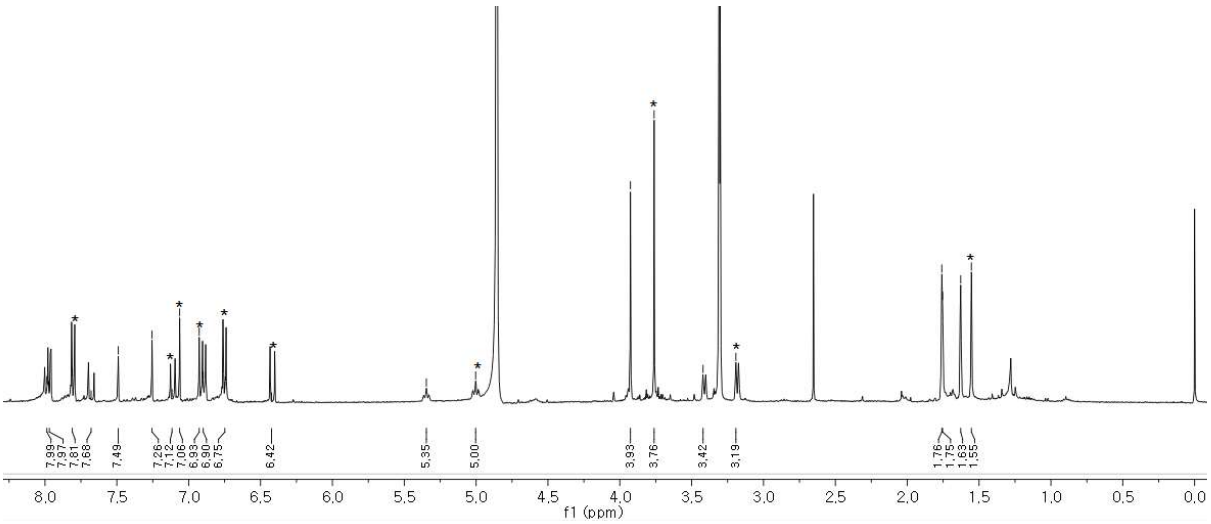

Figure S24. ^{1}H NMR spectrum of metabolite **12** (400 MHz, in CD_3OD). * Signals of *cis* form.

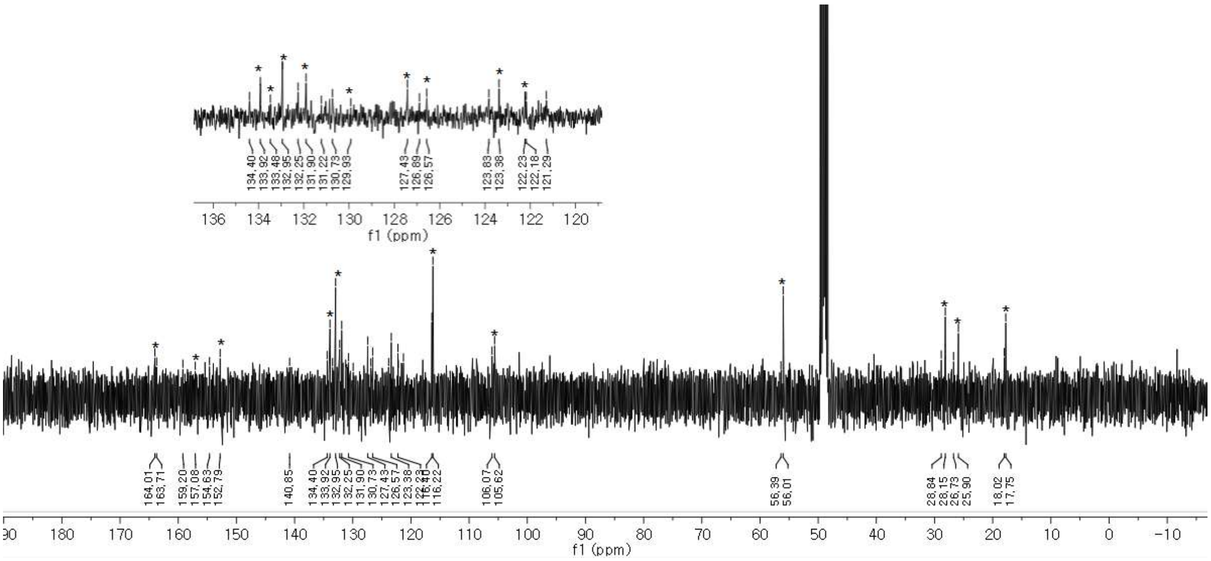

Figure S25. ^{13}C NMR spectrum of metabolite **12** (100 MHz, in CD_3OD). * Signals of *cis* form.

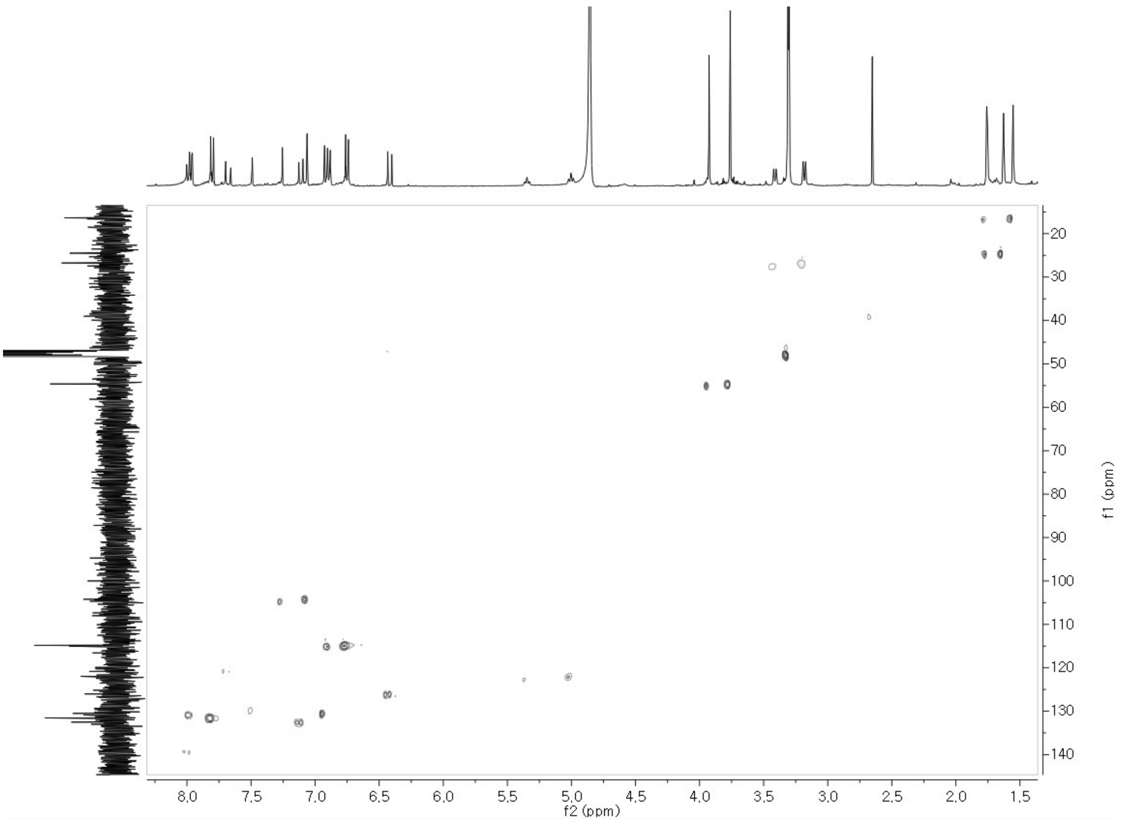

Figure S26. HSQC spectrum of metabolite **12** (400 MHz, in CD_3OD).

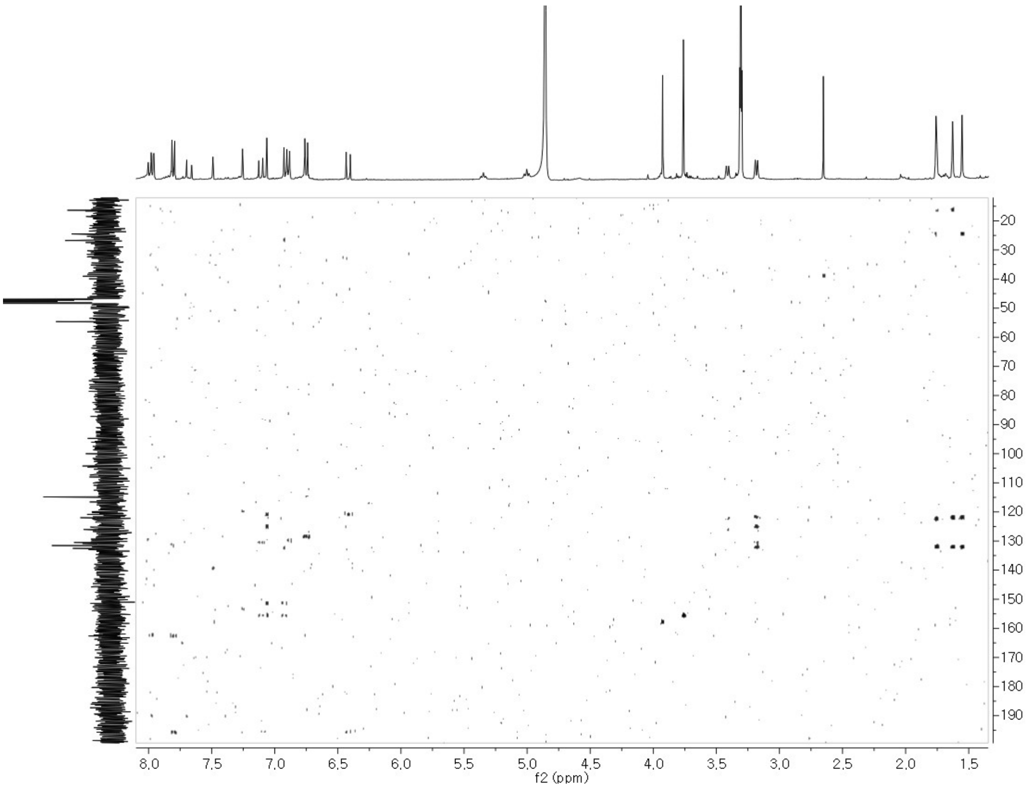

Figure S27. HMBC spectrum of metabolite **12** (400 MHz, in CD_3OD).

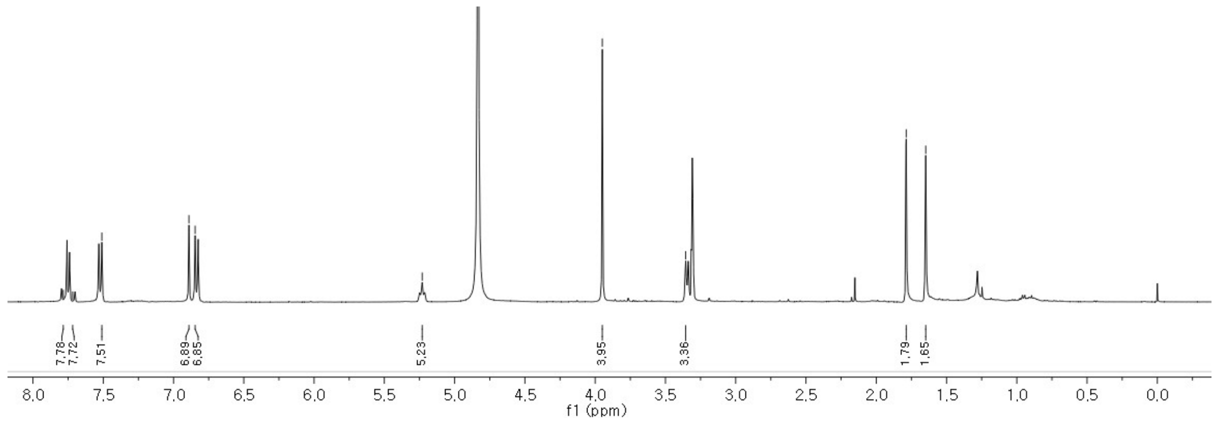

Figure S28. ^1H NMR spectrum of metabolite **13** (400 MHz, in CD_3OD).

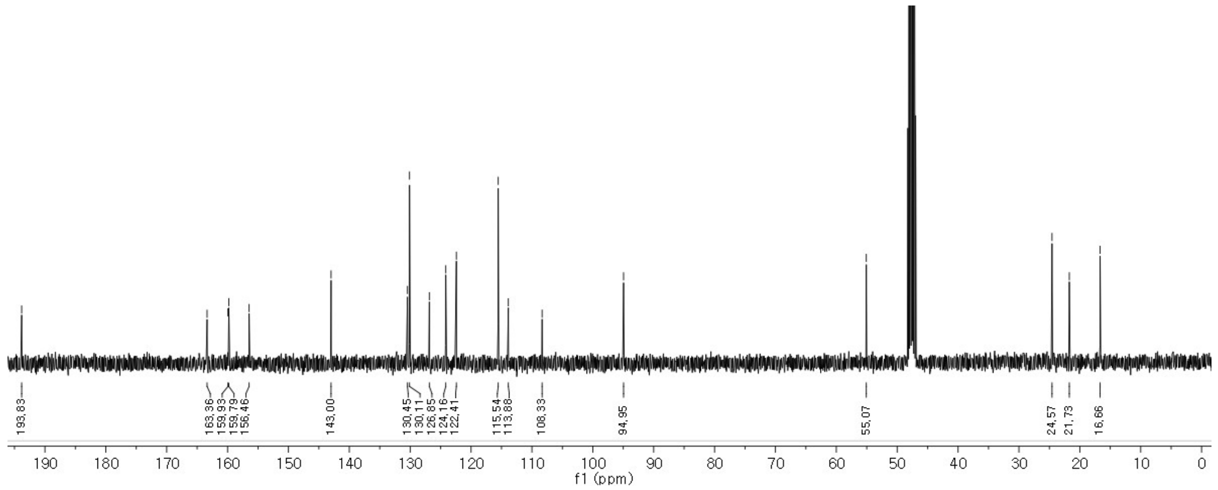

Figure S29. ^{13}C NMR spectrum of metabolite **13** (100 MHz, in CD_3OD).
